# Accelerating the Electrochemical Formation of the δ Phase in Manganese‐Rich Rocksalt Cathodes

**DOI:** 10.1002/adma.202412871

**Published:** 2024-12-23

**Authors:** Tucker Holstun, Tara P Mishra, Liliang Huang, Han‐Ming Hau, Shashwat Anand, Xiaochen Yang, Colin Ophus, Karen Bustillo, Lu ma, Steven Ehrlich, Gerbrand Ceder

**Affiliations:** ^1^ Department of Material Science and Engineering University of California Berkeley CA 94706 USA; ^2^ Materials Sciences Division Lawrence Berkeley National Laboratory Berkeley CA 94720 USA; ^3^ The Molecular Foundry Lawrence Berkeley National Laboratory Berkeley CA 94720 USA; ^4^ Hard X‐ray Scattering and Spectroscopy Program, National Synchrotron Light Source II Brookhaven National Laboratory Upton NY 11973 USA

**Keywords:** battery, cathode, delta phase, disordered rocksalt, electron microscopy

## Abstract

Mn‐rich disordered rocksalt materials with Li‐excess (DRX) materials have emerged as a promising class of earth‐abundant and energy‐dense next‐generation cathode materials for lithium‐ion batteries. Recently, an electrochemical transformation to a spinel‐like “δ” phase has been reported in Mn‐rich DRX materials, with improved capacity, rate capability, and cycling stability compared with previous DRX compositions. However, this transformation unfolds slowly over the course of cycling, complicating the development and understanding of these materials. In this work, it is reported that the transformation of Mn‐rich DRX materials to the promising δ phase can be promoted to occur much more rapidly by electrochemical pulsing at elevated temperature, rate, and voltage. To extend this concept, micron‐sized single‐crystal DRX particles are also transformed to the δ phase by the same method, possessing greatly improved cycling stability in the first demonstration of cycling for large, single‐crystal DRX particles. To shed light on the formation and specific structure of the δ phase, X‐ray diffraction, scanning electron nanodiffraction (SEND) and atomic resolution STEM‐HAADF are used to reveal a nanodomain spinel structure with minimal remnant disorder.

## Graphical abstract

A method of electrochemical pulsing which forms a spinel‐like δ phase in Mn‐rich disordered rocksalt materials with Li‐excess (DRX) (DRX) cathode materials is demonstrated. This concept is extended to large single‐crystalline particles, greatly improving the cycling stability. X‐ray diffraction, scanning electron nanodiffraction (SEND) and atomic resolution STEM‐HAADF were used to reveal a nanodomain spinel structure separated by antiphase boundaries.

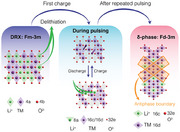

## 1 Introduction

In recent years, disordered rocksalt materials with Li‐excess (DRX) materials have gained momentum as a potential avenue to create more earth‐abundant oxide (or oxyfluoride) cathodes.^[^ ^1^, ^2^, ^3^ ^]^ By utilizing d^0^ transition metals (such as *Ti* ^4 +^ or *Nb* ^5 +^) to lower the disordering temperature,^[^ ^4^ ^]^ and Li‐excess to create percolation paths through the disordered structure,^[^ ^5^ ^]^ materials with very high energy density can be created based on earth‐abundant and low cost redox‐active metals such as Fe, Mn, and Cr.^[^ ^6^, ^7^, ^8^, ^9^ ^]^ Specifically, Mn‐based DRX materials have demonstrated not only very high capacities but also more promising cycling retention. However, the sloping voltage profile,^[^ ^10^ ^]^ relatively slow diffusion,^[^ ^11^ ^]^ and the reliance on high Li‐excess (which often necessitates irreversible oxygen redox^[^ ^7^, ^12^, ^13^, ^14^ ^]^) present challenges to commercial use.

To address these challenges, the concept of introducing partial ordering into DRX materials has gained traction as an approach to flatten the voltage curve and improve diffusivity without relying on a large Li‐excess level. Based on this concept, Mn‐based oxyfluorides with partial spinel‐like ordering can be produced by mechanochemical ball milling when the cation‐to‐anion ratio of the material is between that of spinel (3:4) and rocksalt (1:1).^[^ ^15^, ^16^ ^]^ These materials have demonstrated excellent energy density and rate performance, far in excess of many other ordered and disordered cathodes.^[^ ^17^, ^18^ ^]^ The partial (dis)order observed in these materials effectively eliminates the two‐phase reactions which occur during the full lithiation of ordered spinel, leading instead to solid‐solution behavior.^[^ ^19^ ^]^ However, the use of high‐energy ball milling in synthesis, which is difficult to scale and energy‐intensive, makes practical use of these materials unlikely until a different synthetic method to obtain them is found.

Interestingly, Mn‐rich DRXs, synthesized by more scalable solid‐state methods, have also recently been shown to slowly evolve to a spinel‐like “δ phase” during cycling.^[^ ^20^, ^21^, ^22^, ^23^, ^24^ ^]^ This transformation occurs to the most significant extent in materials with high Mn and low d^0^ ion content, being most prevalent in those compositions with ⩾ 0.6 Mn in the typical rocksalt notation (i.e. ${\mathrm{Li}}_{1.1}{\mathrm{Mn}}_{0.7}{\mathrm{Ti}}_{0.2}O_{2}$).^[^ ^16^, ^25^ ^]^ The formation of spinel‐like order in these materials, evidenced by the evolution of 3 and 4 V plateau‐like features and revealed by XRD, TEM, and NMR, is reminiscent of the cycling evolution of ordered monoclinic and orthorhombic LiMnO_2_, which transform to spinel during cycling.^[^ ^26^, ^27^, ^28^ ^]^ Very recent work on nanosized LiMnO_2_ of both polymorphs has also shown this rapid transformation to spinel, consistent with previous results.^[^ ^29^ ^]^ However, when the δ phase forms in DRX, the transformation appears to be halted before a full conversion to spinel is achieved, possibly by the presence of relatively immobile d^0^ cations (i.e. *Ti* ^4 +^ and *Nb* ^5 +^).^[^ ^30^ ^]^ Previously, it has been argued that this leads to the formation of spinel‐like domains with a short coherence length (<5 nm).^[^ ^30^ ^]^ As a result of the retained disorder, the lithiation remains a solid‐solution process over the 3V plateau, unlike in ordered spinel.^[^ ^19^ ^]^ Further evidence that the material is not a well‐ordered spinel is provided by the sloping 4 V region and the large difference in capacity in the 3 and 4 V region in δ, unlike in a typical spinel.

Despite the promise of the δ phase in addressing many of the remaining challenges of Mn‐based DRX materials, the transformation itself complicates the use of these materials. The initial 20–30 electrochemical cycles required to transform from DRX to the δ phase takes several weeks of cycling at the rates the initial (low Li‐excess) DRX can tolerate.^[^ ^21^, ^30^ ^]^ If the transformation was simply allowed to occur in the early part of an energy storage product or vehicle's lifetime, the drastic changes to the voltage curve, rate capability, and capacity of the transforming material would complicate the ability for battery management systems to accurately monitor and balance cells.^[^ ^31^ ^]^ Additionally, existing DRX materials synthesized by solid‐state methods must be milled during electrode fabrication, which is an energy‐intensive process.^[^ ^32^, ^33^ ^]^ This particle size reduction is required, despite the improved diffusivity of the δ phase product after the transformation, due to the low diffusivity of the DRX material retrieved directly after synthesis,^[^ ^11^, ^30^ ^]^ typically in the range of 10^−15^ − 10^−17^cm^2^ *s* ^−1^.^[^ ^9^, ^34^, ^35^, ^36^ ^]^ Other than the difficulties posed by the milling process itself, the reduction of DRX particles to a size of roughly ≈100 nm necessitates the inclusion of a large content of carbon (often 20%, vs <5% in NMC electrodes) to ensure long‐range electronic percolation through the cathode. As evidenced by efforts to extend the cycle life of similar materials, a larger cathode surface area,^[^ ^37^ ^]^ and especially a larger surface area of additive carbon,^[^ ^38^ ^]^ accelerates electrolyte decomposition during high voltage charging. In general, the larger the particles that can be used, the more stable an electrode material will be in cycling and the less carbon will be needed, so long as the material possesses sufficient electronic and ionic conductivity to tolerate longer transport paths. While DRX materials have thus far been constrained to relatively small particle sizes, the higher‐rate δ phase produced during the transformation may not be so constrained, presenting an interesting possibility for the commercialization of the δ phase‐based DRX.

Synthesis by solid‐state methods often leads to a very wide range of particle sizes, due to the varying growth conditions experienced by different particles.^[^ ^6^, ^39^ ^]^ In past literature, the cycling of large particle DRX at ambient conditions has been claimed, but given the wide distribution of particle size in these solid‐state synthesized samples, it is likely that the small particles present in these electrodes contribute most of the observed capacity.^[^ ^24^ ^]^ It is difficult to avoid the presence of some very large particles (>10 µm) during the solid‐state synthesis of DRX materials, due to the very rapid and inhomogeneous growth of grains at the synthesis temperatures of >1000 °C which Mn‐rich DRXs require.^[^ ^25^, ^36^ ^]^ Therefore, a different synthetic method, capable of producing particles with a more homogeneous distribution of particle sizes in the ≈1 micron range, would be preferable. In previous reports, Chen et al. have produced very uniform single crystalline DRX materials by utilizing a molten salt synthesis technique in which typical solid‐state precursors (Li_2_CO_3_ and transition metal oxides) are immersed in a flux of KCl at ≈950 °C for 12 h.^[^ ^40^, ^41^ ^]^ This allows for the production of single‐crystal particles of ≈5–8 µm, which, while not cycled at room temperature, have been instrumental in characterizing DRX materials.^[^ ^42^, ^43^, ^44^ ^]^ In this work, we have adapted this method to generate DRX materials of more Mn‐rich compositions, with the aim of utilizing the δ transformation to unlock their use.

During the typical formation and aging steps of manufacturing, a Li‐ion cell is charged and discharged slowly at an elevated temperature of 40–60 °C in order to form the solid electrolyte interface (SEI) on the graphite anode and then aged to ensure the cell meets quality standards and has no defects.^[^ ^45^, ^46^, ^47^ ^]^ These processes typically take one to three weeks combined, with formation itself taking a few days, depending on the particular chemistry and protocols used by a manufacturer.^[^ ^46^, ^48^ ^]^ If the required time for the δ transformation could be greatly reduced, it might feasibly be incorporated into or after the cell formation process during cell production. To this end, here we report the rapid transformation of a representative Mn‐rich DRX to the δ phase using an electrochemical “pulsing” method. This electrochemical formation method utilizes an elevated temperature, rate of charge and discharge, and a constricted voltage window to transform DRX material to the δ phase in as little as 1 day, and ≈10 times faster overall. This method was developed for ${\mathrm{Li}}_{1.1}{\mathrm{Mn}}_{0.8}{\mathrm{Ti}}_{0.1}O_{1.9}F_{0.1}$, a representative Mn‐rich DRX which has been previously shown to transform to the δ phase.^[^ ^21^, ^30^ ^]^ The various parameters used in the electrochemical pulsing process are optimized using a series of controlled experiments, leading to insights about the transformation process itself. The method is extended to micron‐sized single‐crystals, a first for DRX materials, demonstrating the generalizability of the technique and allowing for greatly enhanced practicality and cycling stability. The structure of the δ phase, and how this relates to its electrochemistry, are investigated using scanning electron nanodiffraction (SEND), atomic resolution scanning transmission electron microscopy (STEM) high‐angle annular dark field (HAADF), and ex situ synchrotron XRD. Taken together, the results of these measurements confirm that, after pulsing, a nanoscale microstructure of different spinel variants impinging on each other at anti‐phase boundaries has formed. The outcome of this work constitutes a significant step forward in bringing earth‐abundant and energy‐dense Mn‐based DRX materials toward commercial viability.

## 2 Results

### 2.1 Pulsing Method Demonstration

Understanding the mechanism by which the δ transformation occurs, the factors that promote it, and the resulting structure that is produced is essential in order to design an electrochemical formation process for the δ phase. The transformation from a DRX phase to the δ phase requires a concerted rearrangement of most of the transition metals' octahedral positions, as they must organize onto a framework similar to a spinel, with a 16d‐like arrangement. Previous calculations on systems in the Li‐Mn‐O compositional space have demonstrated that in an FCC oxide Mn^2 +^ possesses a significantly lower migration barrier (<400 meV) to hop from an octahedral site into a face‐sharing tetrahedron compared to either Mn^3 +^ or Mn^4 +^, as a result of its symmetric, high spin d^5^ orbital configuration and low octahedral field stabilization.^[^ ^49^ ^]^ Since Mn^3 +^ is prone to disproportionation into Mn^2 +^ and Mn^4 +^, even in cathode materials that nominally utilize only Mn^3 +^/Mn^4 +^ redox, such migration can occur when Mn^3 +^ disproportionates. However, for the barrier to Mn^2 +^ migration to be sufficiently low, the tetrahedron to which a given Mn ion hops must not face share with another transition metal occupied site, and is at its lowest when not even a face‐sharing Li^+^ ion is present.^[^ ^30^ ^]^ As a result, it is expected that Mn migration would be most favorable at high states of charge, when more Li^+^ has been removed from the structure. However, this is at odds with the increased valence of Mn at high states of charge, which leads to less low valent Mn being available to perform the necessary migration hops. As such, one would expect that the transformation occurs mostly during the intermediate voltage parts of cycling, and might be promoted by rapid cycling or “pulsing” in only this region.

**Figure 1:**
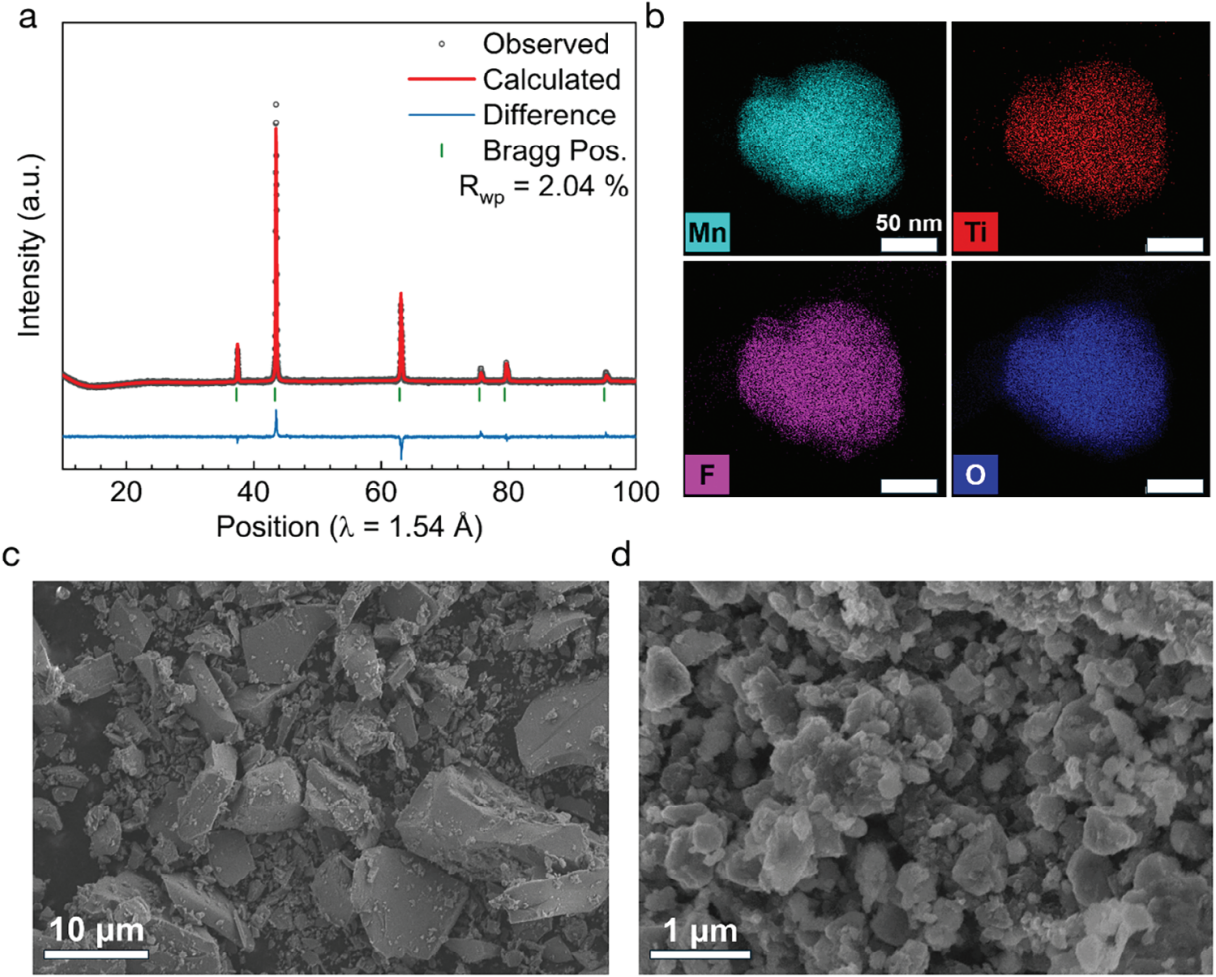
Solid‐state synthesis of ${\mathrm{Li}}_{1.1}{\mathrm{Mn}}_{0.8}{\mathrm{Ti}}_{0.1}O_{1.9}F_{0.1}$. a) XRD pattern and Reitveld refinement for disordered rocksalt structure of solid‐state synthesized ${\mathrm{Li}}_{1.1}{\mathrm{Mn}}_{0.8}{\mathrm{Ti}}_{0.1}O_{1.9}F_{0.1}$. b) EDS mapping performed for Mn, Ti, O and F on these samples after shaker‐milling. c,d) SEM images of the resulting powder before (c) and after (d) shaker‐milling with carbon show that the material initially has a wide distribution of particle sizes (submicron to >10 µm), but that this is reduced to 100–500 nm secondary particles during milling.

In order to test this idea, a representative Mn‐rich DRX, ${\mathrm{Li}}_{1.1}{\mathrm{Mn}}_{0.8}{\mathrm{Ti}}_{0.1}O_{1.9}F_{0.1}$ was synthesized via a solid‐state (SS) method at 1100 °C and subjected to various electrochemical pulsing protocols. The X‐ray diffraction (**Figure** **1**a) and EDS on the as‐synthesized SS large particles (Figure S1, Supporting Information) and shaker‐milled material (Figure 1b) confirm that the material is single‐phase, and the constituent elements are uniformly distributed. As is often the case for solid‐state synthesis, the particle size distribution of the as‐synthesized material is quite wide, ranging from <0.5 µm to >10 µm, as seen in SEM (Figure 1c). However, upon shaker milling with carbon black to prepare a composite for electrode making, the particle size is brought down to a more homogeneous range, varying from ≈100 to 500 nm secondary particle agglomerates (Figure 1d), typical of the carbon composite electrodes produced in previous DRX studies.^[^ ^2^, ^3^ ^]^

In a cathode material which relies exclusively on a single transition metal redox center, one can estimate both the valence of the transition metal and the number of vacancies created by the removal of lithium on charge based on the capacity extracted. While some Mn‐based DRX materials have been shown in previous studies to utilize O‐redox, Mn‐Rich (low Li‐excess) DRX materials show less evidence of O‐redox on account of their larger Mn^3 +/4 +^ redox reservoir.^[^ ^12^, ^24^, ^30^ ^]^ As such, it is expected that in ${\mathrm{Li}}_{1.1}{\mathrm{Mn}}_{0.8}{\mathrm{Ti}}_{0.1}O_{1.9}F_{0.1}$ the amount of charge extracted can be used as a proxy for the vacancy content and Mn valence. To estimate the valence of Mn at various voltages in the first cycle, X‐ray absorption near structure (XANES) spectra were collected at the Mn K‐edge at the typical “full‐charge” voltage of 4.8 V and various points in discharge down to 1.5 V. The selected voltages, along with the Mn absorption edges of the pristine and charged material, along with selected standards, are presented in (Figure S2, Supporting Information). As expected, the pristine material is quite similar to the Mn_2_O_3_ standard and is estimated to have an oxidation state of almost exactly 3.0+ from fitting to the standards (Table S1, Supporting Information). Upon the first charge to 4.8 V, the rising edge shifts most of the way to that of Mn^4 +^ in MnO_2_, giving it a fitted valence of 3.7+. This result corroborates the results of Mn K‐edge XANES on other Mn‐based DRX materials.^[^ ^12^ ^]^ Upon discharge, the average valence of Mn decreases continuously to 3.0+ at 2.0 V, and to just below 3.0+ at 1.5 V. The only deviation from the expected decrease of Mn valence on discharge is the slightly higher valence seen at 4.5 V relative to 4.8 V, which is attributed to slight variations between samples or relaxation before measurement.

**Figure 2:**
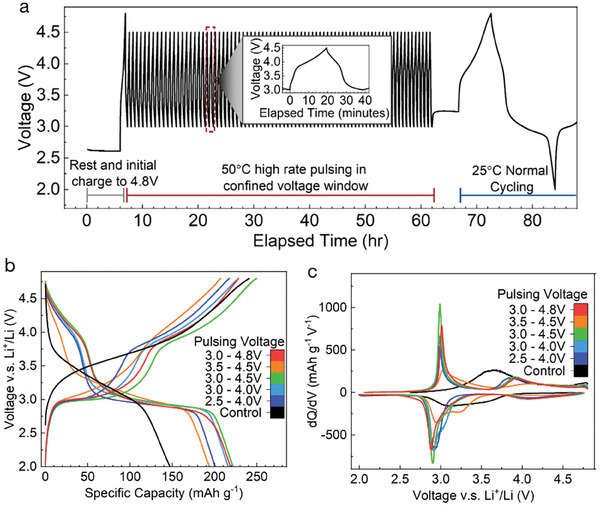
Pulsing protocol and resulting electrochemistry: a) A representative example of the pulsing process of electrochemical formation, showing an initial charge up to 4.8 V, followed by pulsing in a restricted voltage window at high rate and 50 °C, and subsequent normal cycling at 25 °C and 2.0 – 4.8 V. b) The first charge and discharge curve of materials pulsed in various voltage windows at 100 mA g^−1^, 50 °C, for 5 days, along with the pristine material, cycled from 2.0 to 4.8 V. c) Associated differential capacity (dQ/dV) for materials transformed with the same pulsing voltage windows, along with the control, showing different extents of the development of the 3 and 4 V features.

We tested high‐rate, elevated temperature pulsing in various voltage windows for a set time (5 days) and a fixed rate of 100 mA g^−1^ after an initial charge up to 4.8 V. While subsequent cycling was always performed at 25 °C, pulsing was performed at 50 °C, which is within the typical temperature range used during the formation step of cell manufacturing. The full voltage profile of the pulsing process through these three stages of testing is displayed in **Figure** **2**a. Using this elevated temperature was necessary to allow a greater extent of transformation at high rate. For example, in materials regularly cycled for 24 h at 100 mA g^−1^, the transformation was significantly more prominent when cycling occurred at 50 °C rather than 25 °C (Figure S3, Supporting Information). The electrochemistry of cells cycled normally (25 °C, 20 mA g^−1^) after this “pulsing” formation process was evaluated in various voltage windows as shown in Figure 2b with their corresponding dQ/dV plots in Figure 2c. While pulsing in voltage ranges entirely above or below 3.5 V did not seem to transform the material to a large extent (Figure 2b; Figure S4, Supporting Information), those extending upward from ⩽ 3 V transformed to a much greater extent, as evidenced by the formation of the characteristic 3 and 4 V features in the voltage profile of these samples. Of the voltage ranges tested, 3.0–4.5 and 3.0–4.8 V performed almost equivalently, suggesting that little Mn migration occurs in the range of 4.5–4.8 V. In order to minimize electrolyte degradation at the elevated temperature used, 3.0–4.5 V was chosen as the standard pulsing window in the remainder of this work, rather than 3.0–4.8 V.

**Figure 3:**
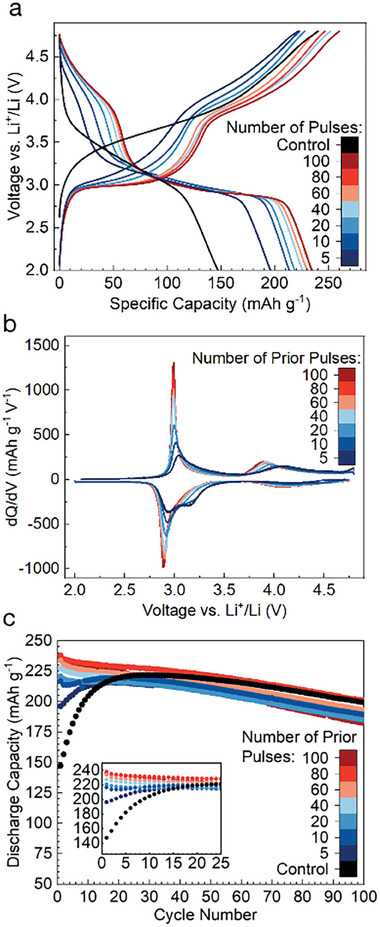
Effect of the number of pulses on the transformation: a) The first full charge and discharge cycles (at 25 °C) of materials pulsed different numbers of times at 100 mA g^−1^, 50 °C, in a window of 3.0–4.5 V along with the pristine material (control), cycled from 2.0 to 4.8 V. b) Differential capacity (dQ/dV) for material pulsed different numbers of times. c) Extended cycling data for material pulsed different numbers of times.

To evaluate the progress of the δ‐transformation and the resulting electrochemistry, we show in Figure 3a the first charge and discharge at 25 °C after a given number of pulses at 100 mA g^−1^ and 50 °C. It can be seen that the capacity of the material rises rapidly, reaching a value of ≈195 mAh g^−1^ in only 5 pulses, requiring less than 1 day of pulsing. At this point, the 3 and 4 V features typical of spinel‐like ordering are already visible, suggesting the formation of spinel‐like environments has begun with only a few pulses. As the number of pulses is increased, these features become more pronounced, with the pseudo‐plateaus becoming both flatter and longer, which can be more clearly observed in the increased height and area of the peaks in the differential capacity curve in **Figure** **3**b. By 60–100 pulses, the changes in the voltage curve have slowed dramatically, with 80 and 100 pulses being almost identical in terms of capacity growth and plateau formation. This cessation of voltage curve changes is taken as evidence that the transformation is “complete,” a point that will be discussed later. The 80 pulses at 100 mA g^−1^ enables the transformation to δ in ≈7 days, rather than the >20 days it takes when DRX is fully cycled at the typical rate of 20 mA g^−1^ used in previous DRX literature. The long‐term cycling of these cells after pulsing, as well as of the control, is shown in Figure 3c. Interestingly, pulsing these materials leads to an increase in capacity, without a corresponding negative effect on subsequent cycle life. The cell pulsed 80 times, for example, possesses a higher capacity than the control throughout the entire duration of 100 cycles.

**Figure 4:**
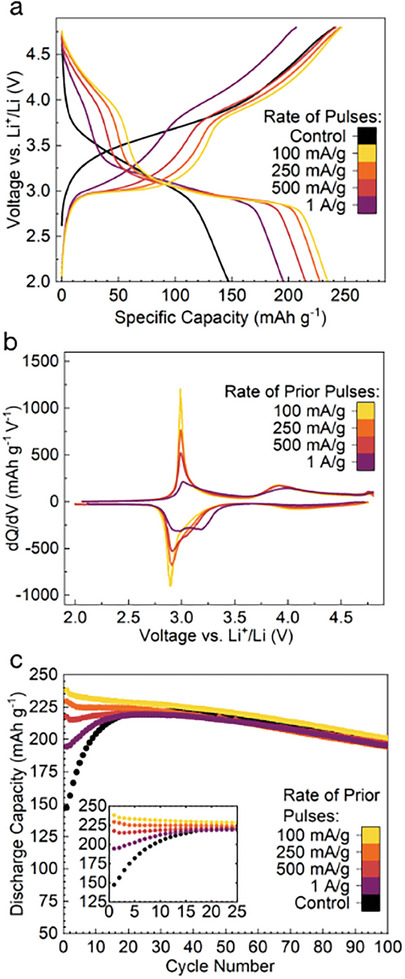
Effect of pulse current rate on the transformation: a) The first charge and discharge curve of materials pulsed 80 times at different rates at 50 °C, in a window of 3.0–4.5 V along with the pristine material, cycled from 2.0 to 4.8 V. b) Differential capacity (dQ/dV) for material pulsed at different rates. c) Extended cycling data for material pulsed at different rates.

Given that 80 pulses at a rate of 100 mA g^−1^ yielded a complete transformation, higher charge and discharge rates were evaluated to determine how much the process could be accelerated. With a fixed number of pulses, going to higher rates decreases the time required for each pulse of a given capacity, as well as the amount of capacity accessed by each pulse, contributing to greatly reduced pulsing duration. As the rate during pulsing is increased from 100 to 200 mA g^−1^, 500 mA g^−1^ and 1 A g^−1^, the extent of transformation and the delivered capacity decreases in the first full cycle, as seen in **Figure** **4**a. The extent to which the 4V feature forms, and the flattening of the 3 V plateau‐like area both decrease when higher rates are employed, as more clearly seen in the dQ/dV profiles displayed in Figure 4b.

While the capacity delivered during pulsing at 100 mA g^−1^ decreases slowly over the course of pulsing, the capacity delivered in a pulse decreases with increasing pulsing rate (Figure S5, Supporting Information). This could suggest that when high pulsing rates are employed, the utilization of material close to the surface of these particles is greater than the bulk, as is often seen in electrode materials at high rates.^[^ ^50^ ^]^ This is also supported by the greater retention of the sloped DRX‐like feature centered at ≈3.2 V in the discharge of materials pulsed at higher rates (Figure 4b), relative to the material pulsed fewer times at a low rate (Figure 3b). These findings suggest that there is a trade‐off between increasing the rate of pulsing to save time, while also ensuring that all the material is being accessed to an extent sufficient to promote the transformation. The long‐term cycling data of these high‐rate pulsed materials is displayed in Figure 4c. As with the material pulsed a different number of times at 100 mA g^−1^, the materials pulsed at high rates retain a comparable or higher capacity than the control throughout cycling. Even the materials pulsed at 500 mA g^−1^ and 1 A g^−1^ deliver capacities of ≈215 and ≈195 mAh g^−1^ in subsequent cycling. This constitutes less than 24 and 12 h of pulsing, respectively, proving that the > 20 days of normal (20 mA g^−1^) cycling taken for this material to transform “fully” to the δ phase, can be shortened dramatically.

### 2.2 Structural and Electrochemical Evolution During Pulsing

Previous studies documenting the δ phase established that the electrochemistry changes during cycling are accompanied by the appearance of new, broad peaks in X‐ray diffraction characteristic of spinel‐like order.^[^ ^20^, ^21^, ^22^, ^23^, ^24^ ^]^ The apparent broadness of these new features in X‐ray diffraction indicated a small coherence length of this ordering, in the range of <5 nm. To better understand when this order forms and how it evolves over the course of cycling, we performed ex situ X‐ray diffraction on electrode materials recovered after pulsing to different extents, as well as after 25 normal galvanostatic cycles over the full voltage range, which is approximately when the capacity of the unpulsed materials peaks.

**Figure 5:**
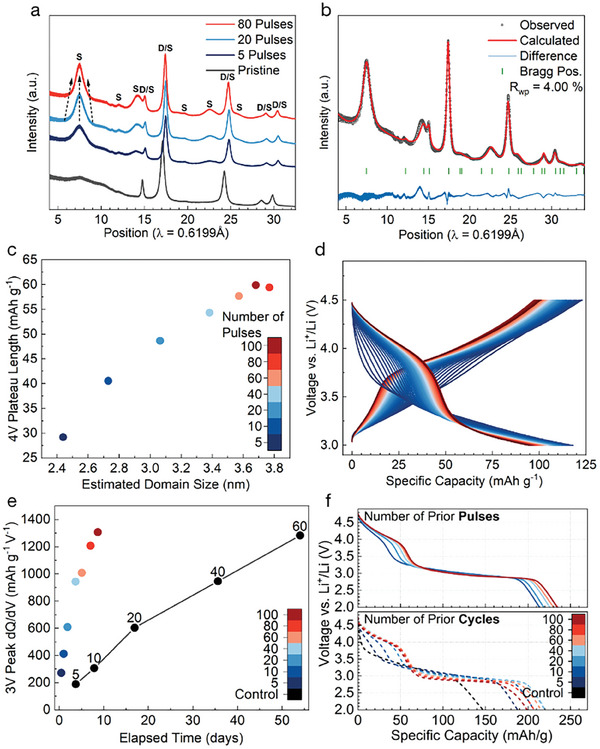
Correlation between structural and electrochemical evolution during pulsing and cycling: a) ex situ XRD pattern of shaker‐milled material in pristine electrode and after pulsing 5, 20, and 80 times. The broad spinel‐like features are denoted with S in addition to peaks that are common to the parent rocksalt lattice and spinel which are denoted with D/S. b) Reitveld refinement of the pattern of material pulsed 80 times using a single phase partially disordered spinel model with 16c/16d disorder and a short coherence length. c) Capacity of the 4V feature after pulsing versus domain size obtained from Reitveld refinement of ex situ samples. d) Evolution of the 4V feature in the voltage curve over the course of 100 pulses (using the same color legend used in Figure 5c). e) Differential capacity (dQ/dV) of the 3V feature as a function of total time in pulsing at 100 mA g^−1^ (colors) and cycling at 20 mA g^−1^ (black). f) Comparison of material pulsed and cycled an equivalent number of times.

**Figure** **5**a shows the ex situ XRD pattern of the pristine, shaker‐milled material, along with material after 5, 20 and 80 pulses (ex situ pattern after other extents of pulsing are presented in Figure S6, Supporting Information). After just 5 pulses, significant and broad spinel‐like features are visible in the pattern (indicated with a S), in addition to the peaks which are common between the DRX and spinel structures (indicated with a D). The S‐labeled peaks not present for the rocksalt structure and are a result from cell doubling, since the spinel unit cell is simply a 2 × 2 × 2 superstructure of rocksalt with a specific cation order. This also explains why only the spinel peaks are broad, as the spinel‐like order can have short‐range coherence, without interrupting the long‐range rocksalt lattice. After pulsing 20 or 80 times, these features become both narrower and more intense. Combined, these changes indicate that the disorder of the spinel‐like environments continues to decrease, while the coherence length increases. This can be interpreted as a growth and continued ordering of spinel‐like “domains” within the material, which decrease the broadness and increase the integrated intensity of these new features, respectively. It is notable, however, just how strong the diffraction features of spinel‐like order are after just 5 pulses, and how the changes decelerate thereafter. Figure 5b displays the XRD pattern of a “fully” transformed electrode after 80 pulses at 100 mA g^−1^. Compared to the pristine material, this material has strong, broad features indicative of significant spinel‐like order. While previous work has claimed that the δ phase contains remnant disordered regions, in addition to spinel‐like order,^[^ ^21^ ^]^ no acceptable refinement could be obtained for a two‐phase model that contains a spinel with a short coherence length. A good fit could only be obtained with a single‐phase model containing only a partially disordered spinel of short coherence length. With a coherence length of roughly 4 nm for the spinel‐like domains, the best fit gives 16c/16d disorder values as low as 2–3%. Higher amounts of 16c/16d disorder are incompatible with the intensity of the spinel‐like diffraction features. Significant inversion of the spinel, arising from 8a occupancy of Mn, is not likely as this would significantly increase the intensity of the (220) peak at ≈12 degrees, which is already well captured with a model possessing only 16c/16d disorder. Because 16c/16d disorder by itself leads only to reduced intensity of the spinel‐like peaks, not broadening, disorder alone cannot explain the low coherence length of spinel‐like order in the δ phase. Since no significant remnant DRX phase is apparent in diffraction, the broadness of the spinel‐like features can only be explained by periodic interruptions to the spinel‐like order, either from disordered “walls” between domains, or antiphase boundaries between spinel domains of different rotational or translational symmetry. In order to further understand the continued structural evolution with pulsing or cycling, the ex situ XRD pattern of samples after various numbers of pulses were fitted with the same model used for the sample pulsed 80 times and displayed in Figure 5b. The details of the Reitveld refinement on additional samples collected after 0, 5, 10, 20, 40, 60, 80, and 100 pulses can be found in the supporting information (Figure S6, Tables S2 and S3, Supporting Information). As the number of pulses increases, the 16c/16d disorder rapidly falls to 2–3%, while the coherence length of the ordering continues to increase from 2.4 to 3.9 nm, between pulses 5 and 100. Between pulses 60 and 100, the changes in domain size slow and halt, in agreement with the lack of change in the voltage curve at this point. The apparent rapid formation of spinel‐like features, combined with the slow increase in coherence length, suggests that the spinel domains form throughout the material in the first few pulses (or cycles) and coalesce to form larger domains over the course of 60–80 pulses before the process stops. Scanning electron nanodiffraction (SEND) performed on material collected after 80 pulses, shows a high degree of order distributed throughout the material, with no remnant disordered regions, in agreement with the XRD refinement (Figure S7, Supporting Information).

In order to understand the interplay between structural and electrochemical changes during the δ transformation, the relationship between the evolution of either the coherence length or the 16c/16d disorder and various features of the electrochemical profile were evaluated (Figure S8, Supporting Information). The clearest relationship is the correlation between the peak differential capacity (dQ/dV) of the 3 V plateau‐like feature on charge and the coherence length (which is taken as an estimate of the domain size). As the estimated domain size in these materials increases, the 3 V feature seen in cycling becomes flatter, leading to a larger peak. Figure 5c shows a similar relationship between the coherence length and the capacity delivered above 3.5 V, providing a simpler proxy for the extent of transformation. Indeed, an equivalent metric can be taken from the capacity of the 4 V region within a pulse itself (although the capacity is slightly lower than in cycling), suggesting that the point at which the transformation stops could be determined during the pulsing program, without the need to execute a normal cycle. The evolution of the voltage curve during pulsing, including the initial growth of the 4 V feature and the cessation of this growth, is shown in Figure 5d. In contrast to the coherence length, the refined 16c/16d disorder falls rapidly to a level <5% and does not appear to correlate with the changes in electrochemistry after ≈40 pulses. The details of these refinements and the estimated coherence length and extent of 16c/16d disorder are presented in Table S2 (Supporting Information). Taking the differential capacity peak of the 3 V plateau as a proxy for the extent of transformation, we can compare the evolution of the control and pulsed materials more easily. In Figure 5e, the peak differential capacity of the control and pulsed materials are plotted, along with the cumulative time taken in either cycling at 20 mA g^−1^ or pulsing at 100 mA g^−1^ to get to that point. While 20–30 cycles at 20 mA g^−1^ takes almost a month, the equivalent extent of transformation can be achieved in 2–3 days of pulsing. Additionally, the 3V dQ/dV feature in material cycled galvanostatically only reaches the amplitude reached by the material pulsed 80 times (7 days) after it has been cycled for over 50 days. Since the pulsing being compared here is at only 100 mA g^−1^, it is expected that future optimization of the pulsing process will accelerate the δ transformation even further.

Figure 5f, shows a comparison between the electrochemistry of the material after being pulsed different numbers of times at 100 mA g^−1^ and the normally cycled control cell after the same number of cycles at 20 mA g^−1^. It is apparent that the formation of the 4 V feature occurs to a similar extent after an equivalent number of pulses/cycles, although the 4 V feature in the control stops growing after ≈40 cycles, while the pulsed material does not do so until ≈60 pulses. This suggests that the effectiveness of a single pulse is roughly equivalent to that of a full cycle, supporting the notion that not all parts of the voltage window contribute equally to the transformation. However, the evolution of the 3 V plateau is quite different in pulsing versus cycling. In the pulsed material, the 3 V plateau becomes flatter and longer, but does not otherwise change significantly after additional pulses. In contrast, the 3 V feature of the cycled material becomes shorter with additional cycles. This shortening of the 3 V feature during cycling, while the 4 V feature continues to increase in capacity, leads the capacity of material to peak after 20–30 cycles, despite the transformation not actually being complete at this point. This might be explained by material degradation or impedance growth which occurs at low voltage, but not in the voltage range used in pulsing (3.0–4.5 V).

### 2.3 Activating Large Single‐Crystal DRX

**Figure 6:**
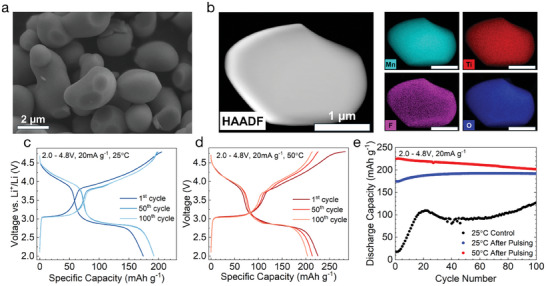
Synthesis and Transformation of Molten Salt Synthesized DRX: a) SEM micrograph of the MS material showing single crystals with a homogenous particle size of 2–3 µm. b) STEM HAADF and EDS performed on the MS sample shows a uniform distribution of Mn, Ti, O, and F in this material. c) Cycling performance of the MS material at 25 °C after 80 pulses with constant voltage holds. d) Cycling performance of the MS material at 50 °C after 80 pulses with constant voltage holds. e) Extended cycling of pulsed MS material at 25 °C and at 50 °C, along with a control cell which has not been pulsed prior to cycling.

Most DRX studies thus far have utilized material extensively milled to produce particle sizes in a range of 100–500 nm. Such high surface area material aggravates any surface‐catalyzed electrolyte degradation and typically requires a large amount of carbon in the electrode to provide electronic contact to all the cathode particles. As previous work has demonstrated improved rate capability of high Mn‐content DRX that has undergone transformation,^[^ ^30^ ^]^ we investigate here whether larger single‐crystalline DRX particles can be activated by pulsing prior to regular galvanostatic operation. To this end, we modified the molten salt method developed by Chen et. al^[^ ^40^, ^41^ ^]^ by increasing the synthesis temperature to 1100 °C and reducing the holding time to 20 min, using a relatively high ratio of 3:1 KCl to precursor, while using the same solid‐state precursors (Li_2_CO_3_ and transition metal oxides) typically employed for the synthesis of DRX. The detailed synthesis procedure is described in the methods section. These conditions allow for the creation of spheroidal single crystal ${\mathrm{Li}}_{1.1}{\mathrm{Mn}}_{0.8}{\mathrm{Ti}}_{0.1}O_{1.9}F_{0.1}$ DRX particles of size 2–3 µm, as shown in the SEM image in **Figure** **6**a. Almost no particles with a diameter of less than 1 micron are observed. The distribution of Mn, Ti, and F observed in TEM‐EDS (Figure 6b), confirms that these elements are uniformly distributed in the material. XRD confirms the product is a phase‐pure DRX (Figure S9).

Because of the dramatically longer diffusion length in these molten salt (MS) synthesized materials (>10 times larger radius), we find that the capacity of the material in each pulse is much lower than for the SS material if a current of 100 mA g^−1^ is employed. This indicates that not all of the material can be accessed in 2–3 µm particles. When a lower current of 20 mA g^−1^ is applied, more capacity is delivered in each pulse, at the expense of the pulsing taking many weeks, and the material achieves a capacity of 184 mAh g^−1^ after pulsing (Figure S10, Supporting Information). This slow and partial transformation of MS DRX particles, even after several weeks of pulsing at elevated temperatures, suggests that an inherent hurdle exists for the transformation of such particles in a reasonable time frame. This problem could in principle be circumvented by using a continuously increasing current density in each subsequent pulse, tracking the increasing rate capability of the material. However, this presents challenges for optimizing the process, as there are numerous choices one has to make about the initial pulsing rate, final pulsing rate, and the rate at which the current is increased. Instead, we attempted to use pulses with constant voltage holds until a lower limit of current is reached (Figure S11, Supporting Information). This allows the initial pulses to proceed more slowly, allowing additional time for the cycling of Li^+^ in the initial DRX material. However, as the transformation proceeds and the diffusivity of the material improves, higher rates would be expected to persist through more of the constant voltage (CV) pulse, leading to high average current densities and an increased average current density in later cycles.

Such a pulsing program utilizing 80 pulses with CV holds at 3.0 and 4.5 V to 10 mA g^−1^ was tested, after an initial charge at 10 mA g^−1^ to 4.8 V. The full pulsing process in this case took roughly 9 days, with a typical pulse cycle of charge and discharge voltage holds lasting for roughly 3 h. The first full charge and discharge cycle of material pulsed in this manner is displayed in Figure 6c, along with the 50th and 100th cycle. As can be seen by the clearly formed 3 and 4 V features, the CV pulsing program successfully transforms the material to the δ phase, providing an initial discharge capacity of 174 mAh g^−1^. The resulting voltage curve of this material is quite similar to that of the smaller particle, shaker‐milled SS material, with two important differences. First, in the MS material, the 3 and 4 V plateau‐like features appear sharper than the smooth features present in the SS material. It is possible that the smoother features of the shaker‐milled SS material result from changes in crystallinity resulting from the shaker‐milling process itself, as previous studies have demonstrated that such milling can introduce defects.^[^ ^51^ ^]^ Second, the lower capacity of the large particle material is mostly due to a lower capacity at the end of the 3 V feature, while the 4 V feature has similar capacity to that seen in the shaker‐milled material.

To further test the stability of this material, cycling was performed where a cell remained at 50 °C after pulsing was complete, as shown in Figure 6d. In the first cycle at 50 °C, the material provides 225 mAh g^−1^ and 700 Wh kg^−1^, demonstrating that a large capacity is still accessible in these large particle materials, even if some capacity is inaccessible at room temperature due to kinetic limitations. Extended cycling of this material at 25 °C in Figure 6e shows some initial capacity gain, after which the capacity remains remarkably stable. Over the course of cycling, the capacity of the material increases from 174 mAh g^−1^ to over 190 mAh g^−1^ before stabilizing, yielding over 600 Wh kg^−1^. This may be due to some small extent of continued transformation during the cycling, which improves kinetics. From cycles 50 to 100, the capacity stays almost constant, with no significant voltage fade or other changes to the voltage curve. The capacity retention at 50 °C is also quite good, with 94.7% and 90.3% of the initial capacity retained at the 50th and 100th cycles, respectively. To confirm that the δ phase formed here is indeed equivalent to that formed in the shaker‐milled SS material, additional synchrotron XRD was performed on MS material recovered after the CV pulsing procedure. The refinement of this XRD pattern (Figure S12, Supporting Information) confirms that this material, like the SS material, possesses a structure with an estimated average spinel‐like domain size of ≈5 nm and a high degree of order (<5% 16c/16d disorder).

### 2.4 The Domain Structure of the δ‐phase

**Figure 7:**
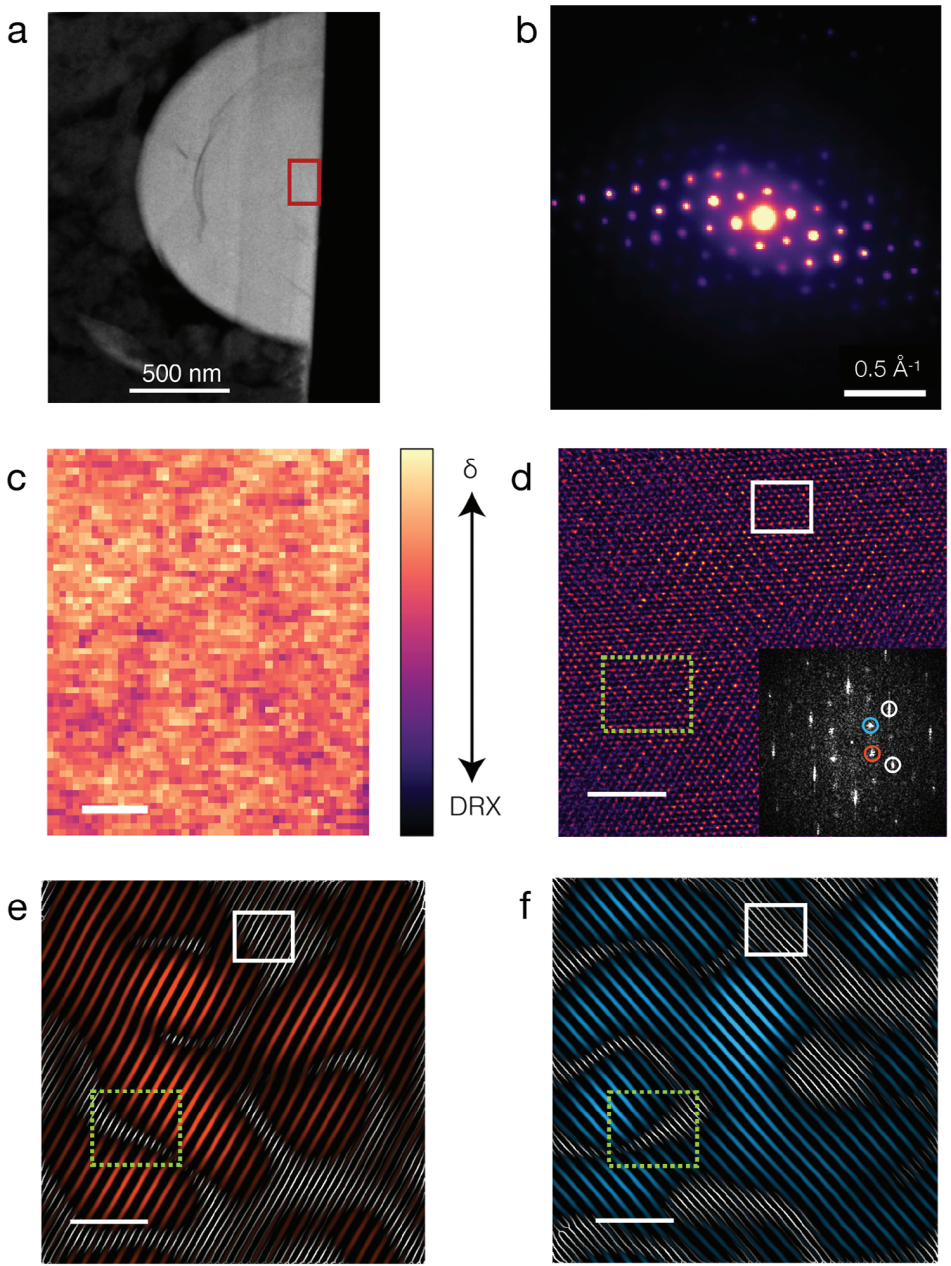
Multimodal structural characterization of the δ phase in the molten salt sample. a) Low magnification STEM‐HAADF image of the pulsed molten salt particle. b) The mean SEND patterns from the region marked in panel a. c) Spatial distribution of the δ phase obtained by virtual imaging the diffraction spots unique to the $\mathrm{Fd}\bar{3}m$ space group (Scale bar: 5 nm) d) Atomic resolution HAADF‐STEM micrograph collected along the [110] zone axis from the region marked in the red box in panel a (Scale bar: 3 nm). The amplitude of its fast Fourier transform (FFT) is shown in the inset, where the Fourier components belonging to the δ phase are marked in red and blue circles. The Fourier components of the parent DRX phase are marked with white circles. The δ lattice obtained by Fourier filtering of each frequency for the red component e) and blue component f) is overlaid on the DRX parent lattice. In panels d, e, and f the white box and the green dashed box mark a disordered region (neither spinel frequency component present) and a translation in the lattice fringes representative of the antiphase boundary respectively.

To understand the transformation of molten salt synthesized DRX particles into the δ‐phase upon pulsing, we conducted scanning electron nanodiffraction (SEND). **Figure** **7**a shows the low magnification HAADF‐STEM image of a typical large molten salt particle (≈2 µm) after pulsing. The mean of the SEND diffraction patterns collected across the red box marked in Figure 7a is shown in Figure 7b. The sharp diffraction spots indicate a high degree of crystallinity of the particle post‐pulsing, and suggest that this particle remains single‐crystalline. Diffraction patterns from selected points have been indexed based on the structural information obtained from the synchrotron XRD refinement of the same samples (Figure S12, Supporting Information) and are provided in Figure S13. We map the extent of δ phase formation (Figure 7c) by integrating the diffraction peaks unique to the δ phase from each of the SEND patterns and normalizing the obtained virtual image across the full acquired scattering range (from 0.175 to 1.2 Å^−1^) to account for the thickness. The radial integration ranges are shown in Figure S14 (Supporting Information) and detailed in the methods section. The integrated virtual image shows a uniform transformation from the DRX to the δ‐phase, with no substantial amount of retained disordered regions.

To investigate the cation ordering in the δ‐phase in detail, we collected an atomic‐resolution HAADF‐STEM micrograph along the [110] zone axis from the region marked in Figure 7a. A low pass Butterworth filtered HAADF‐STEM micrograph is shown in Figure 7d and the raw image is shown in Figure S15 (Supporting Information). The fast Fourier transform of this HAADF‐STEM micrograph is shown in the inset of Figure 7d. The frequency components belonging to the lower symmetry spinel‐like ($\mathrm{Fd}\bar{3}m$) lattice are marked with red and blue circles in the inset of Figure 7d. Filtering the frequency components marked by the red and blue circles, we obtain the lattice fringes shown in Figure 7e,f, respectively. The lattice fringes from the parent DRX phase ($\mathrm{Fm}\bar{3}m$) are obtained by filtering the frequency components marked with gray circles in the inset of Figure 7d. This parent lattice is underlaid as gray lines in Figure 7e,f. The details of the filtering algorithm are provided in the methods section. The spinel lattice fringes in Figure 7e show multiple half‐lattice vector translations, which are representative of antiphase boundaries. One such boundary is marked as a green dashed box in panel d, e, and f of Figure 7. These boundaries are formed when one variant of the spinel ordering impinges on another variant, which is distinct by one of the rotational or translational symmetry operations of the parent rocksalt that are lost in the spinel symmetry. The domain size obtained by counting the average number of the lattice fringes between the antiphase boundaries is estimated to be in the range of 3 to 10 nm.

## 3 Discussion

**Figure 8:**
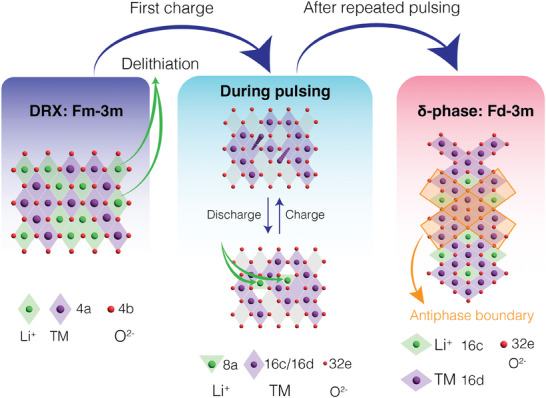
Schematic of δ transformation through pulsing. The accelerated transformation for DRX sample is achieved by a repeated charge and discharge between 4.5 and 3.0 V. This pulsing is carried out at elevated temperature and rate to transform the DRX material into the δ phase more rapidly. The microstructure of δ the phase consists of nanoscale spinel domains which are separated by antiphase boundaries.

The formation of the δ phase upon cycling a Mn‐rich DRX results in a stable cathode material with energy density comparable to Ni and Co‐based NMC cathodes. However, the slow rate at which the transformation proceeds during normal cycling and the associated changes in the voltage profile may pose obstacles to its use in commercial batteries where cycling is unpredictable and irregular. In this work, we show that the transformation to the δ phase can be accelerated via electrochemical pulsing, as summarized in **Figure** **8**. By speeding up the formation of δ from over a month to just days, the transformation may be feasibly integrated into the formation and aging processes of Li‐ion cells. We furthermore demonstrate that this technique can be extended to activate micron‐sized single crystals, thereby negating the need for shaker‐milling DRX with carbon. As demonstrated in this paper, such low surface area materials show near perfect cycling. During pulsing, no capacity or voltage fade is apparent, meaning that the transformation can be promoted without compromising later cycling. Once the δ phase transformation is complete, minimal structural change occurs, in contrast to the voltage fade observed in Li and Mn‐rich layered cathodes.^[^ ^31^ ^]^ The activation of large single crystals, and lack of degradation, bring δ‐DRX significantly closer to commercial viability as an earth‐abundant and inexpensive cathode material.

The δ phase derives it unusual microstructure from the manner by which it forms from the parent DRX rocksalt. The common oxygen sublattice and higher symmetry of a rocksalt allows for multiple possible spinel variants, consistent with the norm of the quotient of the rocksalt and spinel space group. These variants differ by their occupancy of the 16c/16d Wyckoff sites on the $Fd\bar{3}m$ lattice. Our characterization results show that δ consists of small domains of these variants separated by anti‐phase boundaries. It is likely that the formation of δ starts easily in different places in the rocksalt material, independent of its neighboring nuclei, and that these nuclei then grow to impinge on each other.

Our analysis shows that the DRX sample is completely transformed after pulsing, and the resulting microstructure and its electrochemical voltage profile remain remarkably stable. The almost complete extent of δ phase formation after pulsing is evident in the SEND data shown in Figure S7 (Supporting Information) for the SS sample and in Figure 7c for the MS material. The material possesses no obvious large‐length scale (>1 nm) disordered regions, as seen by the predominance of lattice fringes corresponding to the spinel phase ($\mathrm{Fd}\bar{3}m$ space group) in nearly all regions of the HAADF‐STEM micrograph Figure 7e,f. Some remnant disorder is seen in the boundaries of these well‐ordered spinel domains, consistent with the small amount of 16c/16d site disorder required for a good fit of the XRD patterns, but the individual domains appear to be well‐ordered. While the mean diffraction pattern from the SEND image shows sharp peaks indicative that a high degree of crystallinity is retained after δ has formed (Figure 7b), the XRD peaks unique to the $Fd\bar{3}m$ phase (odd *l* index) show selective broadening, indicating that the ordering that introduces these reflections has a small domain size. Estimating the crystallite size from the broadness of these odd l‐indexed peaks^[^ ^52^ ^]^ with the Scherrer equation gives a ≈ 4 nm domain size, consistent with the spinel domains seen in the Fourier filtered atomic‐resolution STEM‐HAADF imaging (Figure 7d,e).

Care must be taken in discerning the extent of (dis)ordering in the δ phase by a visual inspection of a STEM micrograph. Due to the presence of domain boundaries and the overlap of different rotational or translational domains, certain regions can appear disordered upon visual inspection. However, if different phases were present, such as DRX, spinel, and layered, they would be clearly identifiable in the local diffraction patterns in SEND. To distinguish the presence of these phases from the overlapping spinel domains, SEND and HAADF‐STEM measurements have to be carried out in tandem. From our observations, we conclude that the δ phase comprises highly ordered spinel domains separated by thin anti‐phase boundary regions (≈1 nm), and no evidence for a significant fraction of other phases is present.

The efficacy of pulsing in a limited voltage regime to form the δ phase is consistent with our understanding of the kinetics of Mn migration^[^ ^53^ ^]^ and the thermodynamics of the transformation to spinel in Li–Mn–O systems.^[^ ^54^ ^]^ Mn migration is known to be most favorable when Mn is at a low valence and adjacent to tetrahedral sites which do not share any face with octahedra occupied by another transition metal. Such a configuration has been referred to as a 1‐TM (tetrahedral) site. In a Mn‐rich DRX with the composition used in this study, a given tetrahedral site has a 26.6% chance of being adjacent to 1 Mn and 3 Li (a “1‐Mn” site), assuming a random cation configuration. Given this high chance of a tetrahedron being a 1‐Mn site, and the presence of 8 tetrahedra around each Mn, a very large number of potential hops should be available for Mn during the first charge of the DRX structure. This may explain why a significant degree of spinel‐like order is already observed as early as the 5th pulse in this material. As the transformation proceeds, the number of Mn experiencing favorable conditions for migration would be expected to decrease, as the formation of spinel‐like order locks Mn ions into a configuration without such 1‐TM tetrahedron (ordered spinel LiMn_2_O_4_ has only 0, 2, and 4‐TM sites^[^ ^5^ ^]^). Given the decreasing number of opportunities to hop, it is unsurprising that the growth of δ decelerates dramatically and 60–80 pulses through the “effective” window must be carried out to “complete” the transformation.

The voltage window that most effectively forms δ‐DRX can be rationalized from the combined need for low valent Mn and Li vacancies. To allow a low‐energy hop for a Mn adjacent to a 1‐Mn site, the three other octahedral sites which face share with this tetrahedron must be vacated of Li^+^ upon charging, but the Mn valence has to remain below 4+ as Mn^4 +^ shows very little mobility.^[^ ^49^ ^]^ Upon discharge, the octahedral sites must be refilled with Li to destabilize the tetrahedral occupancy of the Mn and push it into another adjacent octahedral sites (or possibly back to the original), in a sort of “musical chairs” mechanism, so that it ultimately can settle on a spinel‐like configuration of octahedral Mn. These conditions require the material to visit a range of Li contents that 1) vacate most octahedra; and 2) promote Li to re‐occupy these octahedra (<3.5 V). These conditions explain several observations from the optimization of the pulsing process. For example, the similarity of material pulsed from 3.0–4.5 and 3.0–4.8 V makes sense given that the average Mn valence is already nearly 4+ at 4.5 V, so there are few Mn^3 +^ to hop into the vacancies created by the last stage of charge. Likewise, it should not be surprising that discharge cutoffs at low voltage, when relatively few octahedral vacancies exist, do not lead to transformation. It may also be noted that the stoichiometry of the material in the effective voltage range is similar to that of a spinel 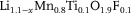 (x ≈ 0.5–0.8). Therefore, it might be argued that this region is effective because it combines a strong driving force toward spinel^[^ ^53^ ^]^ with favorable kinetic conditions for Mn migration.

This development of δ as a uniform, highly ordered, but nanostructured phase might be expected to only be achievable in very Mn‐rich cathodes (such as orthorhombic or monoclinic LiMnO_2_ and DRX), and at low temperature (such as 50 °C). This is because cathodes not rely heavily on Mn redox such as Li and Mn‐rich oxides (${\mathrm{Li}}_{1.2}{\mathrm{Ni}}_{0.2}^{2+}{\mathrm{Mn}}_{0.6}^{4+}O_{2}$) there is not enough low valent Mn present, and Mn^3 +^ is only available at the end of the discharge when octahedral vacancies are limited. These materials instead experience slow voltage fade and a very slow conversion from layered to a more spinel‐like structure. While the transformation in Mn‐rich DRX is much more rapid than in the Li–Ni–Mn–O layered system, the evidence presented here suggests that the transformation in DRX is halted at a smaller domain size and perhaps with more retained disorder than in previous work on orthorhombic and monoclinic LiMnO_2_.^[^ ^28^ ^]^ It is important that the transformation does in fact stop at a smaller domain size, as continued transformation to a fully ordered spinel would risk creating a two‐phase reaction on the 3 V plateau, as is seen in these ordered materials which transform to spinel.^[^ ^55^ ^]^ Indeed, recent theory work^[^ ^19^ ^]^ has argued that some disorder in a spinel is critical for turning the 3 V plateau into a solid‐solution. DRX may be predisposed toward smaller domains due to several factors. The cation disorder in DRX statistically creates a large variety of local environments, and those that are “spinel‐like” may act as fast nucleation sites for forming the spinel domains, unlike in well‐ordered LiMnO_2_ compounds which have no local spinel‐like environments. A second factor is that the high symmetry of DRX creates multiple crystallographically distinct variants which are expected to have equal average nucleation rate. This is in contrast to ordered, LiMnO_2_ in which the reduced symmetry will favor a subset of the spinel variants. Nucleating fewer variants will inevitably lead to fewer antiphase boundaries, as the nucleated domains grow during pulsing/cycling until they impinge on each other. A final factor in limiting the domain size may be the presence of immobile Ti^4 +^ in DRXs, which would inhibit the growth of these domains as it cannot easily move between octahedral sites.^[^ ^30^ ^]^ Thus, despite the near complete local conversion to spinel, the domains in the δ phase derived from DRX are limited to only several nm, instead of >10 nm seen in transformed orthorhombic and monoclinic LiMnO_2_.^[^ ^28^ ^]^ The use of temperatures much higher than 50 °C to promote the transformation may risk continued coalescence of the domains, and therefore a two‐phase reaction at 3 V, in addition to increased electrolyte breakdown. We find slightly higher capacity with the use of 50 °C for pulsing than with 60 °C for shaker‐milled material (Figure S19, Supporting Information).

The material's electrochemical behavior only superficially resembles that of a macroscopic spinel. The 4 V region in δ is not a plateau as in spinel and has less capacity than the 3 V region (unlike in spinel where they are equal in capacity). Previous work on cycling‐induced δ‐like material provided evidence from in situ XRD that the two‐phase region near 3 V present in a regular spinel is replaced by a gently sloping solid‐solution in δ.^[^ ^30^ ^]^ The voltage profiles shown in this work similarly do not show a plateau near 3 V, only a sloped voltage indicative of solid‐solution. Hence, it seems that even with well‐ordered spinel domains, a solid‐solution can be achieved as long as those domains are small enough, consistent with general ideas in statistical mechanics that only extended systems can create a first order phase transition.

We observe in XRD that when the molten salt material is fully discharged after pulsing some of the diffraction peaks of the cubic structure split, indicating a potential collective Jahn–Teller distortion (Figure S16, Supporting Information). It is possible that this Jahn–Teller distortion contributes to the reduced diffusivity at the end of discharge seen in GITT and rate tests (Figures S17 and S18, Supporting Information). It appears that the domain structure does not eliminate the collectiveness of the JT effect when most Mn is +3. Nonetheless, this JT distortion does not create a compositional two‐phase region, which is the main reason for the degradation of crystallite integrity seen in ordered spinel when cycled over the 3 V plateau.^[^ ^56^ ^]^ It is possible that by modifying the domain structure, the distortions could be delayed enough to allow improved rate performance. Such improvements seem especially likely considering that the diffusivity estimated by galvanostatic intermittent titration (GITT) obtained for the pulsed material is already significantly improved, ≈10^−14^cm^2^ *s* ^−1^, rather than the range of 10^−16^–10^−15^cm^2^ *s* ^−1^ typical of DRX materials.

As shown in this first demonstration of micron‐sized δ phase, this material can deliver over 700 Wh kg^−1^ at 50 °C, and reasonable cycle life. While there have been no cell‐level safety tests performed on DRX, the thermal stability of δ at the top of charge is likely to be good as a result of the high stability of Mn^4 +^, relative to Ni^4 +^ in layered cathodes, making cell‐to‐pack benefits similar to those achieved for LFP likely.^[^ ^57^ ^]^ With a typical crystal density near 4 g cm^−3^, DRX also provides a higher density than LFP. This combination of good intrinsic energy density and likely thermal stability may make δ‐DRX competitive with high‐energy Ni‐rich packs without the cost and environmental issues of nickel.

The observation that essentially no degradation to the material occurs during pulsing at elevated temperature (and perhaps more generally in the moderate voltage window of 3.0–4.5 V), also indicates that pulsing may be a “no compromise” way of activating these materials prior to normal cycling. The existence of degradation primarily at low voltage indicates an interesting inversion of the traditional state of charge (SOC) best practices that the battery field has learned from layered materials. In Ni‐rich cathodes, the high‐voltage part of the cycling window is by far the most problematic for cycle life.^[^ ^58^ ^]^ In the δ phase, the Jahn–Teller distortions at the end of discharge may make the high voltage region the “safer” operational window. While it is true that above 4.5 V the electrolyte is almost certainly being oxidized at the active carbon or cathode surface in the composite cathode.^[^ ^59^ ^]^ this material provides negligible capacity above 4.4 V, meaning the upper cutoff can likely be lowered substantially from the 4.8 V employed here. As such, δ phase cells may be more typically operated in at high states of charge 60–100% charged region, unlike Ni‐rich chemistries which rapidly degrade when cycled to full charge.

We recommend that all those who study these materials utilize the pulsing method as a standard formation protocol, so that the community can characterize and study the cycling stability of equivalent, demonstrably fully transformed, end‐state δ. As demonstrated in this work, the 20–30 cycles typically taken before the capacity of a Mn‐rich DRX peaks is simply the point at which capacity fade in shaker‐milled materials tends to outpace the continually slowing transformation to the δ phase. At least for this prototype composition ‐ ${\mathrm{Li}}_{1.1-x}{\mathrm{Mn}}_{0.8}{\mathrm{Ti}}_{0.1}O_{1.9}F_{0.1}$ ‐ we now expect that in both cycled and pulsed materials, the transformation takes 60–80 pulses/cycles to complete. Without using this method to promote the complete conversion to δ before further evaluating the material, there is a real danger that the literature will become confused with characterizations of degraded, partially transformed materials.

The potential to use as‐synthesized micron‐sized materials, and perhaps also to reduce the amount of carbon (which is typically introduced during milling) are expected to decrease the cost of composite cathode production for DRX materials. The improved cycling stability of these large particles may also considerably decrease the amount of specialized particle and electrolyte engineering which may be required to attain long cycle life. Additionally, the use of the 1.5–4.8 V voltage window, often used in the literature, must also be reigned in to avoid excessive electrolyte oxidation and reduction, with which excellent cycling stability in full cells with practical loading and lean electrolyte is not possible. Eliminating the use of lower cutoff voltages below 2 V will also eliminate the low voltage tail seen in many of these materials. We see the goal of attaining a thermally safe and truly stable, Mn‐based cathode with ≈200 mAh g^−1^ and >600 Wh kg^−1^ at reasonable rates, large particle size, and low carbon loading as the target toward which researchers studying the δ phase should guide their focus. If the pulsing method is adopted as standard with this aim in mind, the focus of research on Mn‐based DRX materials can be redirected from the details of the first 20 cycles, to performance well beyond the first 100.

## 4 Conclusion

In this work, we have demonstrated a method of electrochemical pulsing which is intended to serve as a formation process for the in‐situ formation of the δ phase from Mn‐rich DRX. We extended this method from the traditional shaker‐milled DRX particles on which it was developed to micron‐sized material produced using an adapted molten salt method. The production of reliable end‐state δ as large single‐crystalline particles, has allowed for more detailed characterization of the fine domain structure in this material using HAADF‐STEM, SEND and ex situ XRD. Combining these methods, we have shown that the δ phase is composed of nanometer‐sized domains that meet at antiphase boundaries, and that these domains achieve a very high degree of order, without any significant remnant disordered regions. The remaining rate capability and minor stability issues at low voltage are pointed out as perhaps the two most important outstanding issues in these materials. It is hoped that the common adoption of the pulsing process will ease future research to better understand and improve this promising class of energy dense and earth‐abundant materials.

## 5 Experimental Section

### Material Synthesis

The solid‐state material was synthesized using a traditional solid‐state method. Li_2_CO_3_ (Sigma Aldrich, 99.9%), Mn_2_O_3_ (Alfa Aesar, 99%), TiO_2_ (Sigma Aldrich, 99.9%), and LiF (Alfa Aesar, 99.9%) were used as precursors. All precursors were mixed in stoichiometric portions with ethanol in a Retsch PM 200 planetary ball mill at a rate of 250 rpm for 12 h. A 10% excess of Li_2_CO_3_ was added to compensate for possible loss during synthesis at 1100 °C. The precursors were dried in an oven at 70 °C overnight before being pelletized. In the case of molten salt synthesis, the same precursor was mixed with KCl (Sigma Aldrich, 99%) by hand for 10 min in a 1:3 Precursor:KCl weight ratio before being pelletized. In both the case of molten salt and solid state synthesis, pressed pellets were heated to 1100 °C at a rate of 10 °C min^−1^ under argon flow in a tube furnace, and held at this temperature for 20 min before natural cooling to room temperature. For the molten salt material, the heated pellet was washed directly with deionized water twice, and ethanol once, before drying at 70 °C overnight under vacuum. For solid state material, the produced pellets were ground by hand prior to shaker‐milling with carbon. All powders were transferred to and stored in an argon‐filled glovebox. SEM images of as‐synthesized and shaker‐milled powders were collected using a Zeiss Gemini Ultra‐55 Analytical Field Emission SEM.

### Electrochemistry

All cathode films were composed of active materials, Super C65 (Timcal), and polytetrafluoroethylene (PTFE, DuPont, Teflon 8A) in a weight ratio of 70:20:10. Prior to cathode fabrication, the solid state material was shaker‐milled with the Super C65 for 1 h under argon atmosphere using a SPEX 800M mixer/mill. The molten salt material was mixed with Super C65 by hand in a mortar and pestle for 20 min. Both the hand‐mixed molten salt and shaker‐milled solid‐state mixtures with carbon were mixed manually with PTFE in a mortar and pestle. The mixture was then rolled into thin films inside a glovebox. The active material loading density of all films was ≈3–4 mg cm^−2^. Commercial 1 M LiPF_6_ in ethylene carbonate (EC) and dimethyl carbonate (DMC) solution (1:1 volume ratio) was used as the electrolyte. A glass microfiber filter (Whatman) was used as the separator. FMC Li metal foil was used as the anode. Coin cells were assembled inside the glovebox and tested on an Arbin battery test instrument. The specific capacities were calculated based on the weight of active materials (70%) in the cathode films. Pulsing was conducted in multi‐zone temperature chambers connected to the same Arbin test instruments, which were set to either 25 or 50 °C, and cycled by combining typical galvanostatic or potentiostatic program steps. Molten salt‐synthesized samples were pulsed using an initial constant current step at +/‐ 10 A g^−1^ until the cutoff of either 4.5 or 3.0 V was reached, and then a constant voltage hold was employed until the current dropped to +/‐ 10 mA g^−1^. After pulsing, cells were directly switched to cycling at 25 °C. For the galvanostatic intermittent titration (GITT) measurements, each step in the voltage profiles corresponded to a galvanostatic charge/discharge of 10 mAh g^−1^ at a rate of 20 mA g^−1^ followed by a 6 h relaxation step.

### Ex Situ Synchrotron XRD and XAS

Synchrotron XRD and XAS patterns were measured at Beamline 7‐BM of the National Synchrotron Light Source II (NSLS‐II). In order to avoid the diffuse diffraction feature of PTFE coincident with the (111) peak of spinel‐like order, ex situ cathode films for XRD were prepared by mixing the active materials, Super C65 and PVDF in at a weight ratio of 7:2:1 in NMP and casting onto aluminum. The resulting electrode film was dried overnight before assembly into cells to perform pulsing/cycling. After pulsing/cycling, cells used to produce ex situ samples were held at a desired voltage for 6 h. The cells were then disassembled and the cathode film was washed with diethyl carbonate (DEC). Cycled PVDF films for XRD were peeled off the aluminum to allow packing into Kapton capillaries. PTFE films used for XAS were sealed in Kapton tape for transport and measurement.

All synchrotron XRD refinements were carried out using the TOPAS software package. A single‐phase spinel structure model was used when refining the XRD patterns of cycled and pulsed electrodes. The reflections generated by the spinel‐like order, resulting from those with an odd l index, were selectively broadened as previously reported for δ and γ‐LiFeO_2_ type ordering.^[^ ^60^ ^]^ The broadening factor used on the spinel‐like peaks was used in the Scherrer equation to estimate the coherence length of the domains, assuming a shape factor of 0.9. Ex situ XAS measurements at the Mn and Ti K‐edge were performed in a transmission mode on films identical to those used in electrochemical measurements. The energy calibration was accomplished by simultaneously measuring the spectra of Mn and Ti metal foil. Data processing was carried out with the Athena software package.^[^ ^61^ ^]^

### Transmission Electron Microscopy Sample Preparation

To prepare ex situ samples for SEND and HAADF‐STEM characterization, intact electrodes identical to those used for electrochemical tests (molten salt synthesized, pulsed 80 times) were collected after pulsing and washed with diethyl carbonate (DEC) and transferred under argon. A thin lamella suitable for transmission electron microscopy (TEM) imaging was prepared using the dual‐beam focused ion beam (FIB) method, utilizing the Helios G4 UX instrument, housed at the National Center for Electron Microscopy (NCEM), Berkeley. To protect the sample, 100 nm thick Pt was deposited on the surface using a 5 kV electron beam and a beam current of 1.6 nA before lift out. Subsequently, 1.5 µm Pt was deposited on the top of the electron beam deposited Pt using a Ga‐ion beam at 30 kV and 0.26 nA. After a lamella was lifted out from the cathode film, it was welded to a FIB grid using Pt. The lamella was thinned down to ≈ 1 µm using a Ga‐ion beam with 30 kV accelerating voltage and 0.75 nA current. To prevent beam damage subsequent thinning to ≈100 nm was carried out using a 5 kV accelerating voltage and 0.15 nA current. A final thinning step was carried out using a 2 kV ion beam. To remove the amorphous layer and thin further to achieve regions with thickness ≈50 nm, a low energy Ar‐ion beam at 900 eV was used for 8 mins in a nanomill.

### Scanning Electron Nano Diffraction (SEND)

The SEND patterns were collected using a ThemIS 60‐300 microscope equipped with a Gatan K2 detector housed at NCEM, Berkeley. The indicated semi‐convergence angle was 0.62 mrad. The SEND patterns were analyzed using the py4DSTEM package version 0.14.8.^[^ ^62^ ^]^ To reconstruct a virtual image for the spatial extent of the spinel phase, only the peaks unique to the spinel phase were integrated. These radial ranges are marked in the Figure S14. The scattering angles for these peaks are (0.175, 0.250) Å^−1^, and (0.59, 0.67) Å^−1^. The integrated virtual image was normalized for thickness by integrating the region between the scattering angles from 0.175 to 1.2 Å^−1^.

### Atomic Resolved STEM‐HAADF

The atomic resolution STEM‐HAADF imaging was performed using the TEAM I microscope (double aberration‐corrected Thermo Fisher Scientific Titan 80–300 kV) housed at NCEM, Berkeley. The microscope was operated at 300 kV with an indicated convergence angle of 30 mrad. The particle was tilted to [110] zone axis before atomic resolved imaging. The raw HAADF image is shown in Figure S15 (Supporting Information). An adaptive local thresholding of 51 pixels is performed in MATLAB to remove any non‐uniform illumination in the image. To remove high‐frequency noise a third order low pass Butterworth filter with a frequency cut‐off of 160 pixels is applied in the frequency domain.^[^ ^63^ ^]^ The filtered STEM‐HAADF image is shown in Figure 7d.

The Bragg filtering was done to identify the spinel lattice by filtering for the two spinel peaks in the FFT of the STEM‐HAADF micrograph, shown in the inset of Figure 7d. The STEM‐HAADF image is first windowed using a Tuckey (or tapered cosine) window. The application of this window reduces the spectral leakage and edge artifacts thereby enhancing the accuracy of subsequent frequency‐based analysis. Following this, filtering masks are applied to the spinel frequency components in the Fourier space and inverse Fourier Transformed to obtain the spinel lattices in real space. Only half of the spinel peaks are considered for the filtering since the conjugate points contain the same information in a Fourier transform of a real‐valued image through Hermitian symmetry.^[^ ^64^ ^]^ The spinel lattices shown in Figure 7e,f are color‐based on the spinel peaks marked in the FFT of the STEM‐HAADF micrograph. That is, the filtering of the spinel peak marked in the FFT by a red circle gives Figure 7e and filtering the spinel peak marked in the FFT by a cyan circle gives Figure 7f. Following a similar approach, the parent DRX lattice was overlaid on the obtained spinel lattice. The frequency components corresponding to the respective DRX lattice in the Fourier space are marked as gray circles in the FFT pattern shown in the inset of Figure 7d. These mathematical filtering operations were achieved using an inhouse MATLAB script.

### Energy Dispersive X‐Ray Spectroscopy

Energy‐dispersive X‐ray (EDX) spectroscopy elemental mappings were obtained using an FEI TitanX 60–300 transmission electron microscope housed at the NCEM facility, Berkeley. The spectral maps were collected at 300 kV accelerating voltage, Bruker‐FEI Super‐X four quadrant detector. The different elemental maps were drawn using Mn‐kα, Ti‐kα, O‐kα, and F‐kα peaks. The EDS data was analyzed using the Bruker esprit software.

## Conflict of Interest

A patent application has been filed for the pulsing process described in this work.

## Supporting information

Supporting Information

## Data Availability

The data that support the findings of this study are available from the corresponding author upon reasonable request.
