# Supplementary material for: Accelerating the Electrochemical Formation of the δ Phase in Manganese‐Rich Rocksalt Cathodes
Source: Adv Mater. 2024 Dec 23;37(6):2412871. doi: 10.1002/adma.202412871 (PMC11817910; doi:10.1002/adma.202412871)
Supplement: Supplementary file 1 — Supporting Information [file ADMA-37-2412871-s001.pdf]

ADVANCED MATERIALS

Supporting Information

for *Adv. Mater.*, DOI 10.1002/adma.202412871

Accelerating the Electrochemical Formation of the δ Phase in Manganese-Rich Rocksalt Cathodes

*Tucker Holstun, Tara P Mishra, Liliang Huang, Han-Ming Hau, Shashwat Anand, Xiaochen Yang, Colin Ophus, Karen Bustillo, Lu ma, Steven Ehrlich and Gerbrand Ceder**

Supplementary information for accelerating the Electrochemical Formation of the δ Phase in Manganese-Rich Rocksalt Cathodes

Tucker Holstun[1,2,*], Tara P Mishra[1,2,*], Liliang Huang[2], Han-Ming Hau[1,2], Shashwat Anand[2], Xiaochen Yang[1,2], Colin Ophus[3], Karen Bustillo[3], Lu Ma[3], Steven Ehrlich[4], Gerbrand Ceder[2]

[1]Department of Material Science and Engineering, University of California, Berkeley, CA, USA
[2]Materials Sciences Division, Lawrence Berkeley National Laboratory, Berkeley, CA, USA
[3]The Molecular Foundry, Lawrence Berkeley National Laboratory, Berkeley, CA, USA
[4]Hard X-ray Scattering & Spectroscopy Program, National Synchrotron Light Source II, Brookhaven National Laboratory, NY, USA
[*]Contributed equally to this work
gceder@berkeley.edu

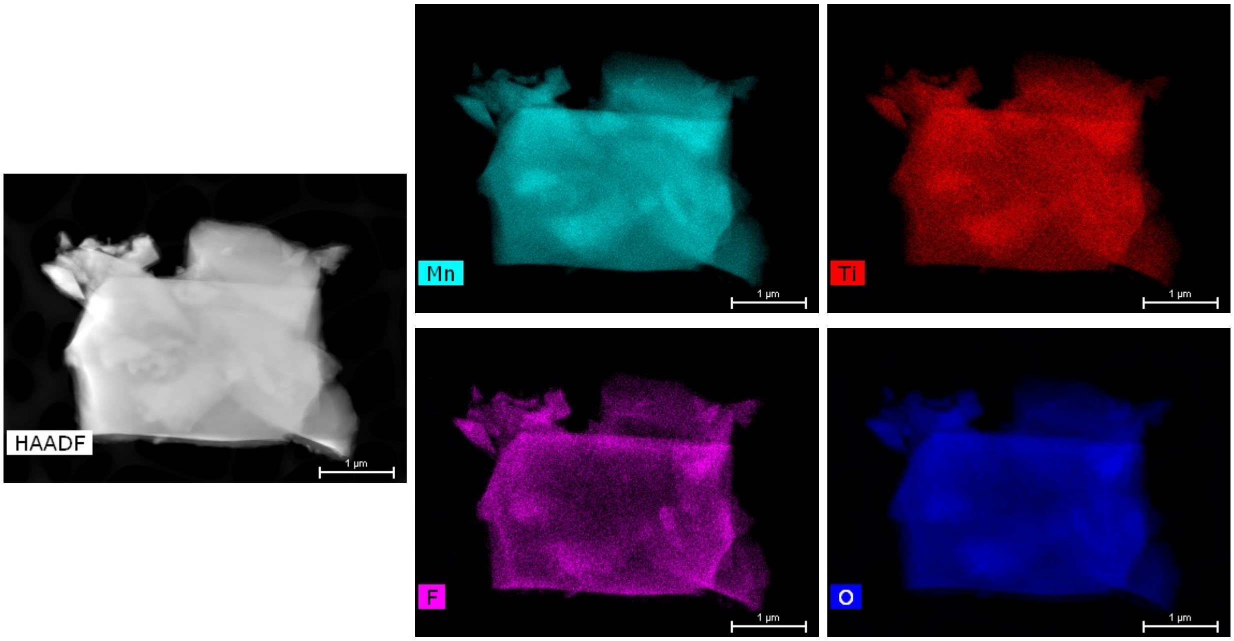

Figure S1: EDS of as-synthesized solid state material. Mapping performed for Mn, Ti, O and F on large particles retrieved directly from solid state synthesis.

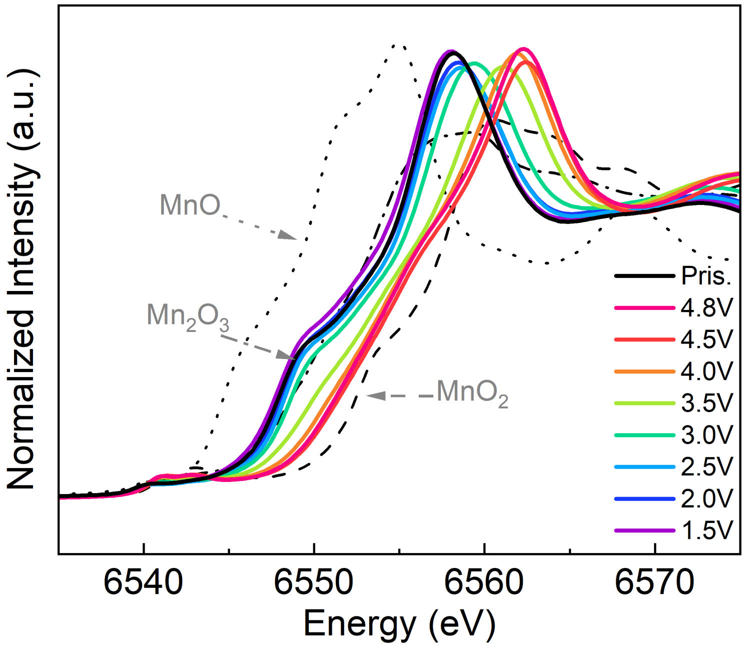

Figure S2: *Ex situ* Mn K-edge XANES measurements. XANES spectra measured at the Mn K-edge of pristine solid state material discharged to various voltages after an initial charge to 4.8 V.

Voltage (V)	Estimated Mn Valence
4.8	3.67
4.5	3.73
4.0	3.62
3.5	3.49
3.0	3.17
2.5	3.04
2.0	3.00
1.5	2.95

Table S1: Estimated Mn valence from XANES at various voltages. Estimated Mn valence of the solid state synthesized material upon discharge to different voltages, as obtained by linear combination fitting of the Mn K-edge XANES spectra using manganese oxide standards.

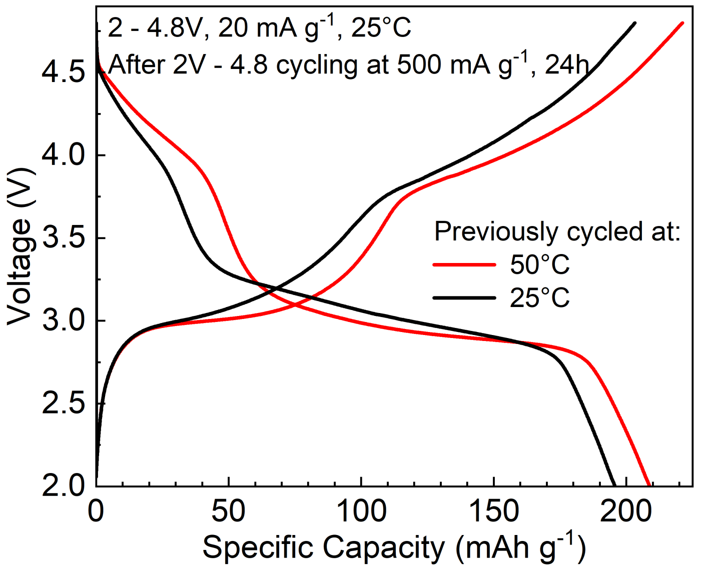

Figure S3: The efficacy of accelerating the transformation to δ by employing elevated temperature during cycling. The first full cycle at 25°C and 20 mA g^{-1} after fixed-time cycling for 24 hours at the high rate of 500 mA g^{-1} at both 50°C and 25°C.

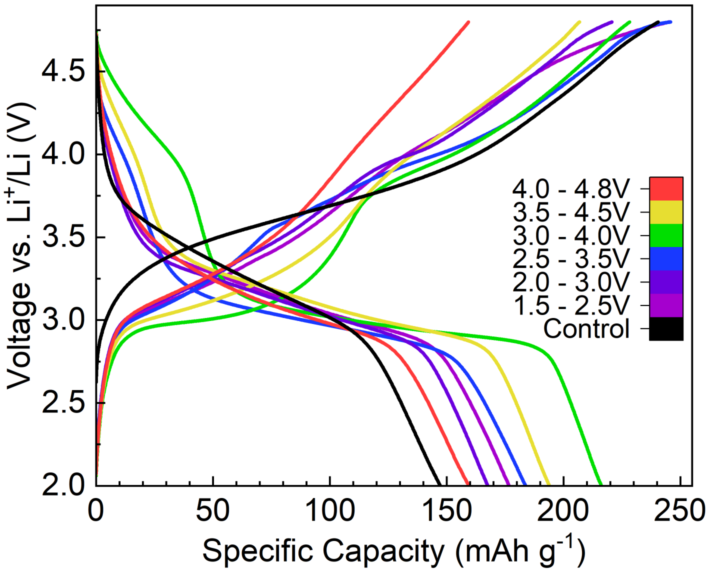

Figure S4: The efficacy of pulsing in different narrow voltage windows. The first full cycle at 25°C and 20 mA g^{-1} after fixed-time cycling for 5 days at the moderate rate of 100 mA g^{-1} at 50°C in a various voltage windows.

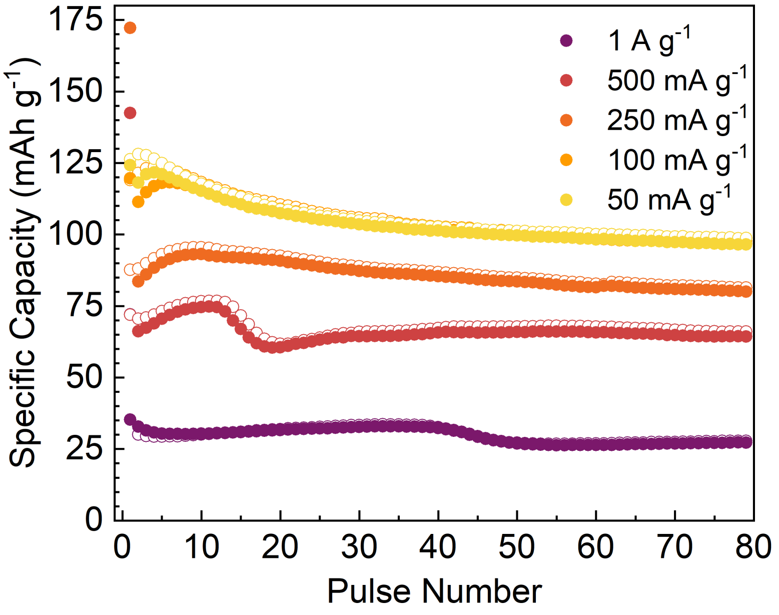

(a)

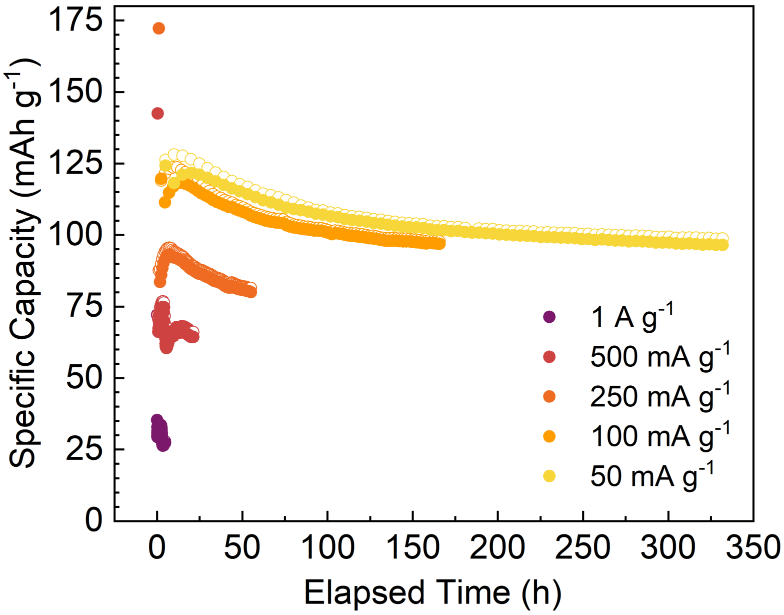

(b)

Figure S5: The time efficacy of pulsing at different rates. (a) Capacity delivered vs. pulse number and (b) Capacity delivered vs. elapsed time during pulsing of shaker-milled solid state material 80 times at 50°C, from 3.0 V to 4.5 V at different rates.

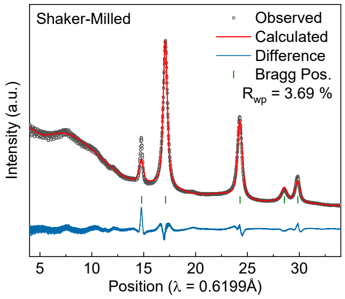

(a)

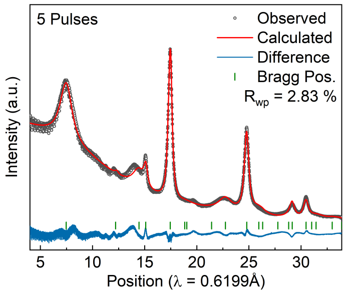

(b)

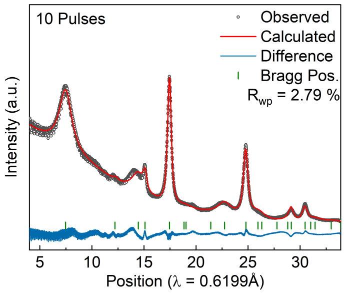

(c)

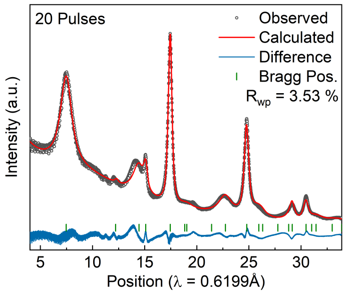

(d)

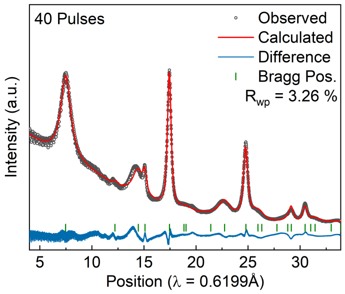

(e)

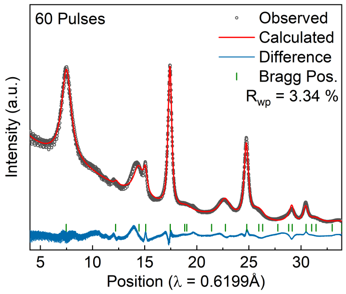

(f)

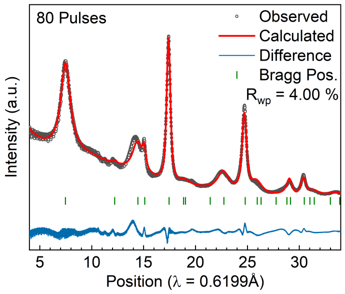

(g)

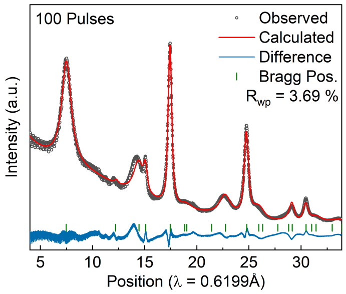

(h)

Figure S6: Synchrotron XRD of solid state material. XRD Rietveld refinements for shaker-milled solid state material subjected to different numbers of pulses. Pristine shaker-milled material measured as a powder (a), while all others measured for electrodes measured *ex situ* (b-h).

Space group: Fm-3m; a = b = c<br>$[Li/Ti/Mn]_{4a}[O/F]_{4b}$						
Atom	Wyckoff Position	x	y	z	Occupancy	B_{iso}
Li	4a	0	0	0	0.55	0.5
Mn	4a	0	0	0	0.4	0.5
Ti	4a	0	0	0	0.05	0.5
O	4b	0.5	0.5	0.5	0.95	0.5
F	4b	0.5	0.5	0.5	0.05	0.5

(a)

Space group: Fd-3m; a = b = c<br>$[Li]_{8a}[Mn/Ti]_{16c}[Mn/Ti]_{16d}[O/F]_{32e}$						
Atom	Wyckoff Position	x	y	z	Occupancy	B_{iso}
Li	8a	0.125	0.125	0.125	1	0.5
Mn/Ti	16c	0	0	0	x	0.5
Mn/Ti	16d	0.5	0.5	0.5	0.9-x	0.5
O	32e	0.27011	0.27011	0.27011	0.95	0.5
F	32e	0.27011	0.27011	0.27011	0.05	0.5

(b)

Table S2: Structural parameters used for all refinements. Structure, atomic positions, occupancies, and temperature factor used to refine (a) pristine and shaker-milled DRX, and (b) ex-situ pulsed samples.

Sample	Refined Mn/Ti 16c Occupancy (%)	16c/16d Disorder (%)	Coherence Length of spinel-like order (nm)	R_{wp} (%)
Pristine	45	100	-	4.90
5 Pulses	4.6	10.2	2.44	2.83
10 Pulses	3.6	8.0	2.73	2.79
20 Pulses	2.6	5.9	3.06	3.53
40 Pulses	2.1	4.5	3.38	3.26
60 Pulses	1.6	3.6	3.57	3.34
80 Pulses	2.1	4.5	3.77	4.00
100 Pulses	2.0	4.5	3.68	3.69

Table S3: Results of Rietveld refinements. Refined structural parameters for shaker-milled solid state material subjected to different numbers of pulses.

Supplementary Note: δ Refinement. The table above summarizes the refinement results for the preceding series of materials pulsed different numbers of times. The model used, as outlined in the methods section, is derived from that in [60] and the same as that used in [30]. In this model, a single phase spinel with 16c/16d site disorder is used. Selective broadening is applied to only peaks with an odd index - those originating from spinel-like order. This selective broadening is then used with the Scherrer equation to calculate the implied coherence length, or domain size, of the spinel-like order. The 16c/16d site disorder is applied by treating Mn and Ti as equivalent, due to their near indistinguishability to XRD. After a single refinement, the atomic positions were fixed for all subsequent refinements. Li is assumed to only occupy 8a sites, and no other ion is assumed to occupy 8a. This model produces good fits of the δ, while a single-phase model cannot.

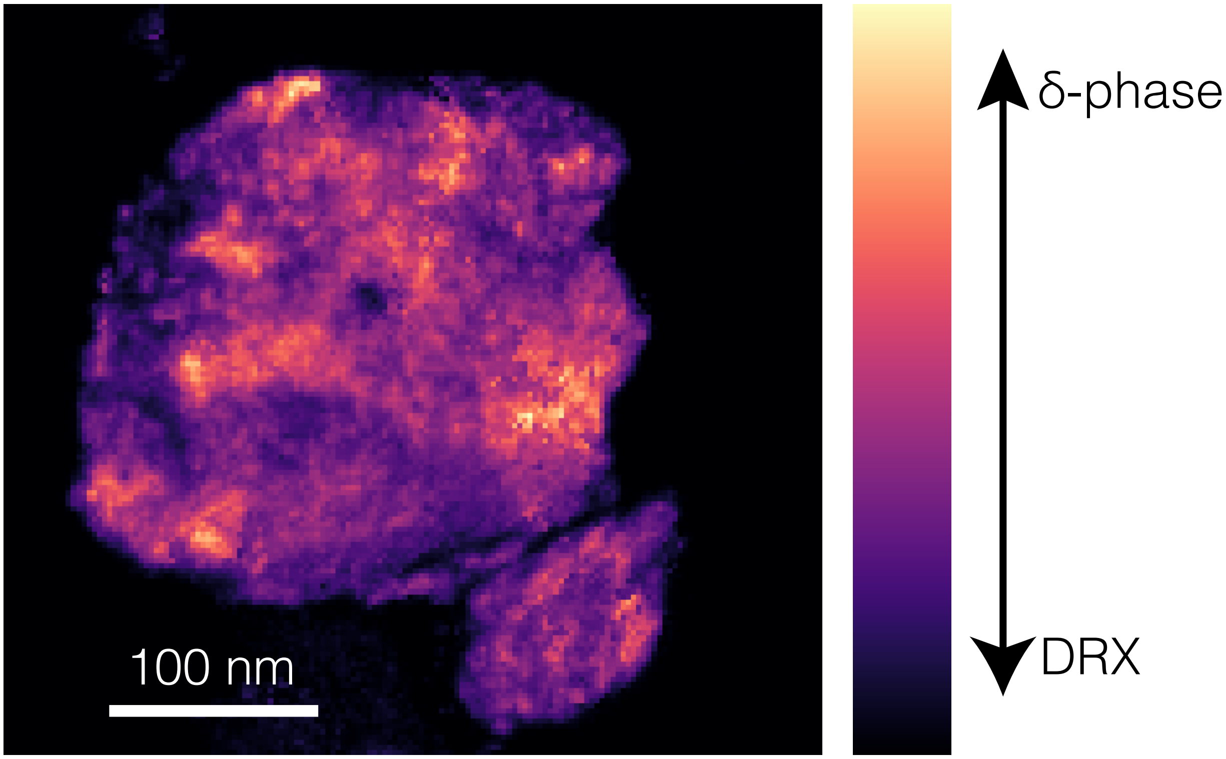

Figure S7: SEND mapping performed on pulses solid state synthesized material. Spatial distribution of the δ phase obtained by virtual imaging the diffraction spots unique to the Fd$\bar{3}$m space group.

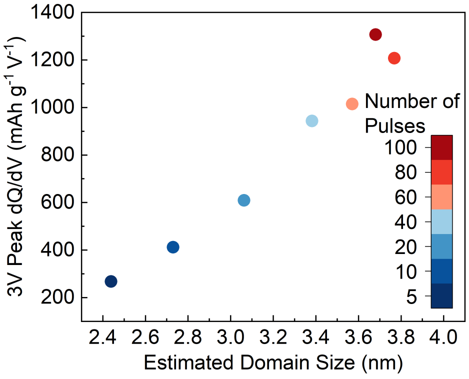

(a)

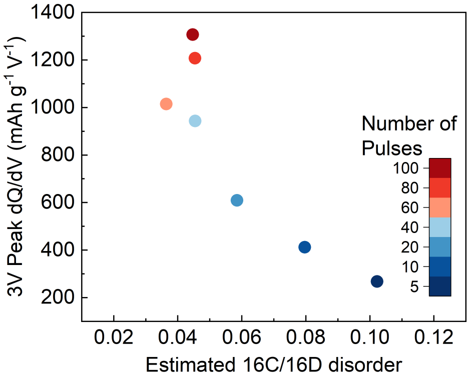

(b)

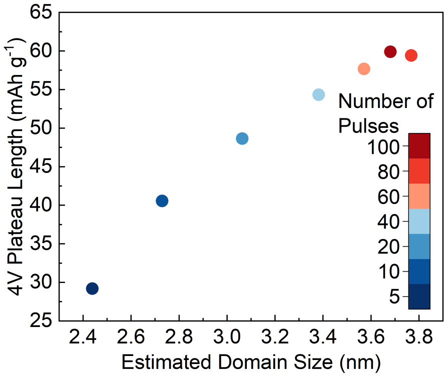

(c)

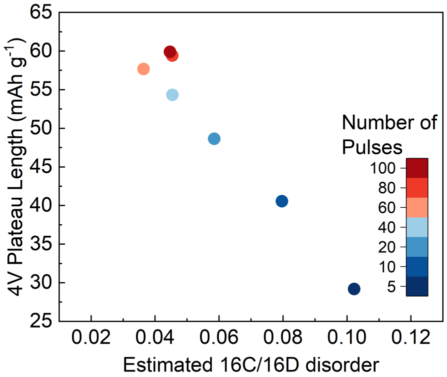

(d)

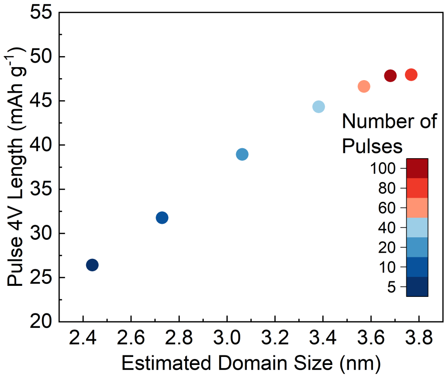

(e)

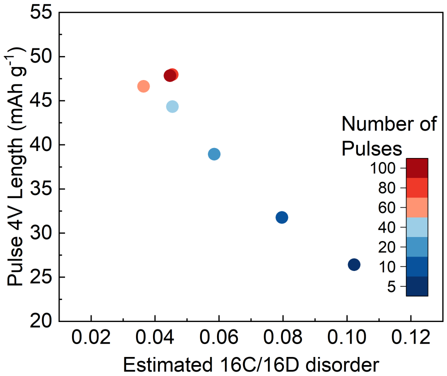

(f)

Figure S8: Correlation between electrochemical change, domain growth, and ordering in δ. (a) Peak differential capacity of 3V plateau after pulsing vs. estimated domain size, (b) Peak differential capacity of 3V plateau after pulsing vs. estimated 16C/16D site disorder, (c) Length of 4V plateau after pulsing vs. estimated domain size, (d) Length of 4V plateau after pulsing vs. estimated 16C/16D site disorder, (e) Length of 4V plateau during pulsing vs. estimated domain size, (f) Length of 4V plateau during pulsing vs. estimated 16C/16D site disorder.

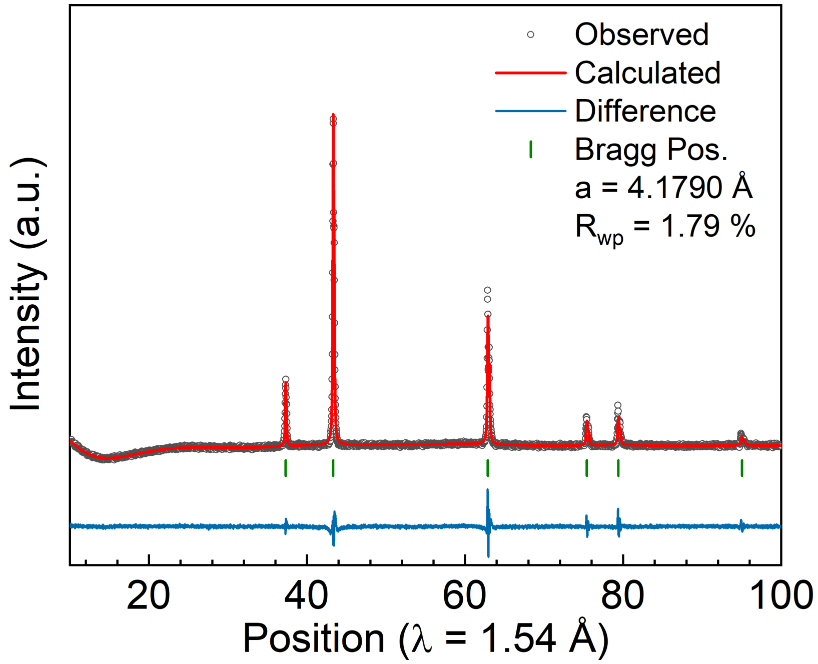

Figure S9: Bench top XRD of molten salt material. XRD Reitveld refinement for as-synthesized molten salt material.

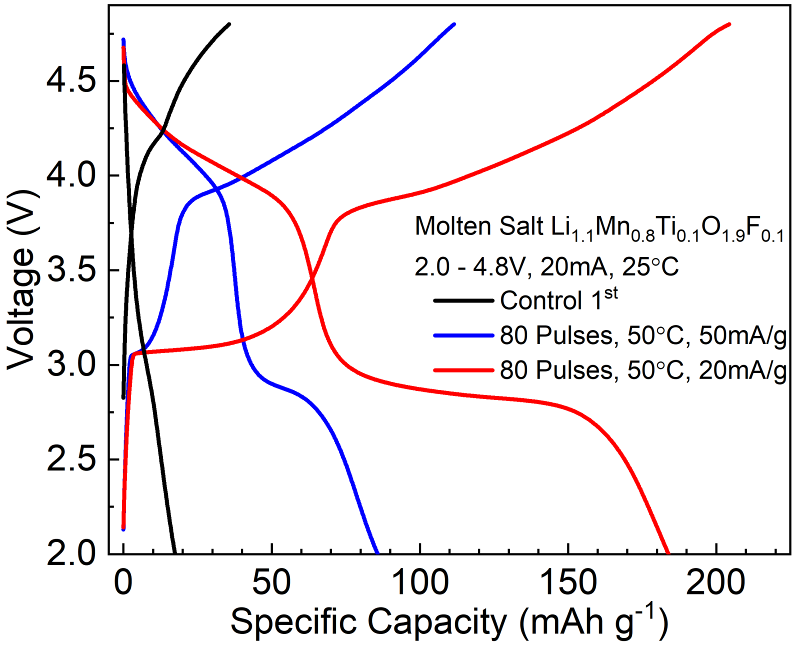

Figure S10: Application of constant-current pulses to molten salt material. First full cycle of molten salt material electrodes pulsed at 50°C and low constant-current rates, along with the pristine material.

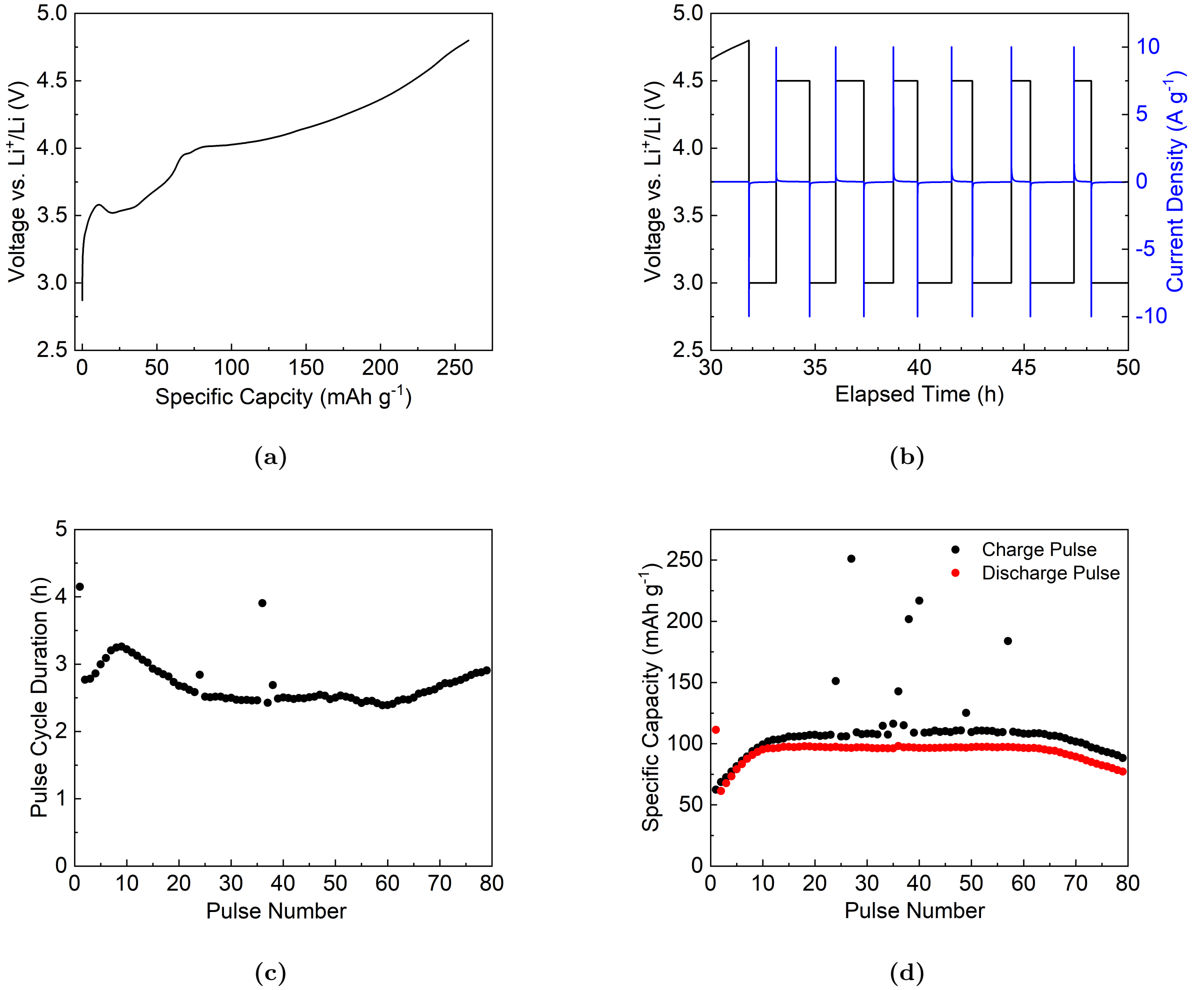

Figure S11: Application of constant-voltage pulses to molten salt material. (a) First charge of molten salt material electrode at 50°C and 10 mA g^{-1}. Decrease in voltage at roughly 3.5 V attributed to large overpotential at low SOC in large particle DRX. (b) Voltage and current profile of constant voltage cycling from 10 A g^{-1} down to 10 mA g^{-1}. (c) Duration of each pulse over the course of 80 such pulses. (d) Specific capacity of each charge and discharge pulse. Higher charge capacities attributed to electrolyte oxidative events, which temporary approach or exceed 10 mA g^{-1} during a voltage hold at 4.5 V and 50°C.

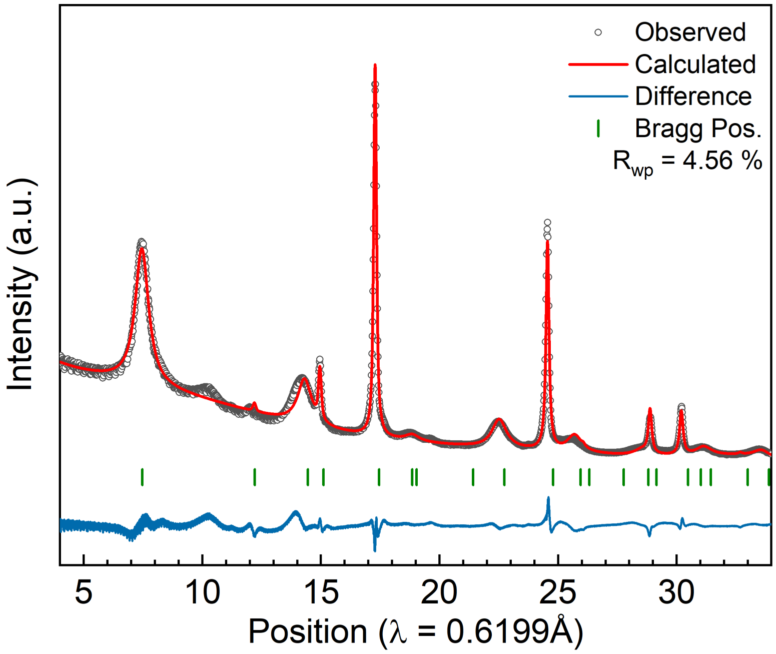

Figure S12: Synchrotron XRD of transformed molten salt material. XRD Reitveld refinement for molten salt material measured *ex situ* after pulsing using same model as for shaker-milled material. The additional peak at roughly 10 degrees is attributed to the diffuse diffraction of carbon in the electrode

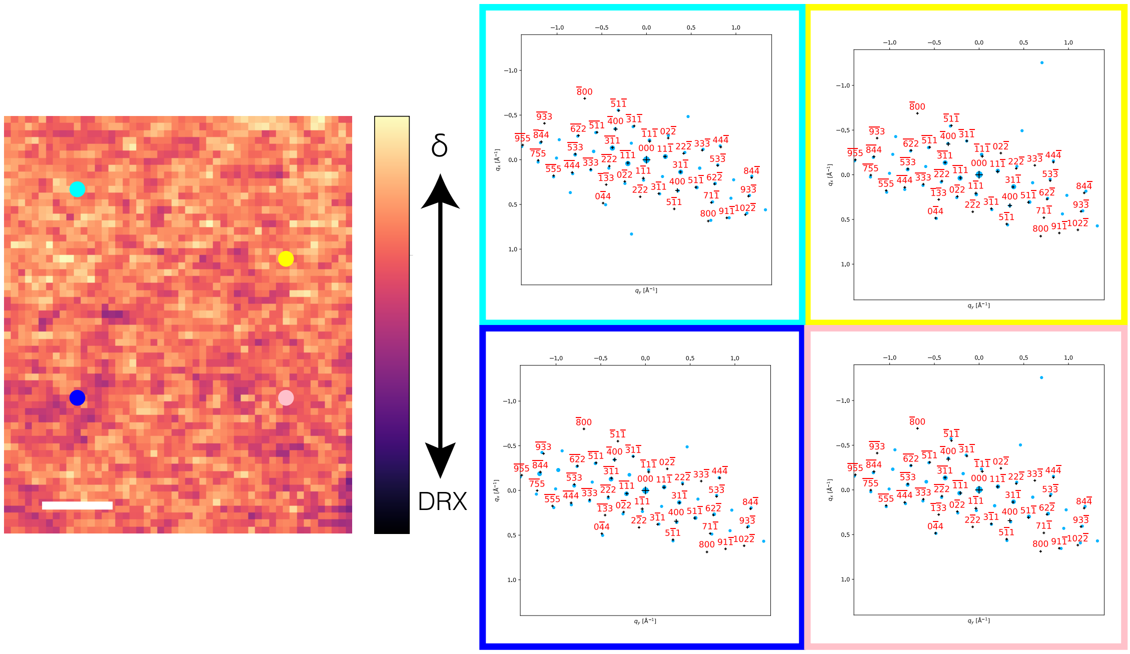

Figure S13: Selected SEND diffraction patterns. The best fit zone axis to these selected diffraction patterns were found to be [0.045 0.643 0.764].

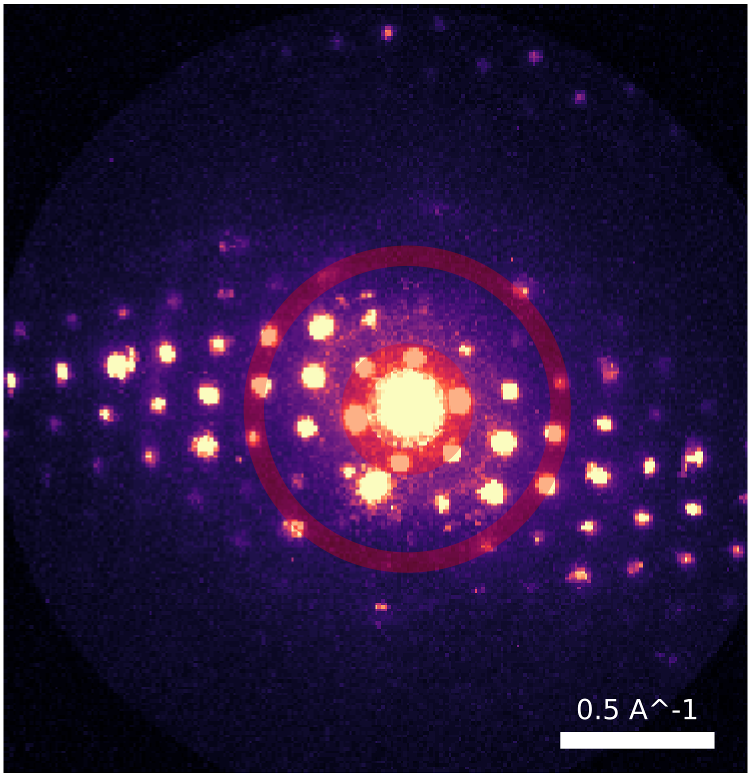

Figure S14: Marked scattering range for δ phase virtual imaging. The scattering angles peaks unique to the spinel space group Fd$\bar{3}$m which are used for the virtual imaging as shown in Fig. 6(c) of main text are marked as annular red circles. These the inner and outer scattering vectors of these peaks are: (0.175, 0.250) Å^{-1}, and (0.59, 0.67) Å^{-1}.

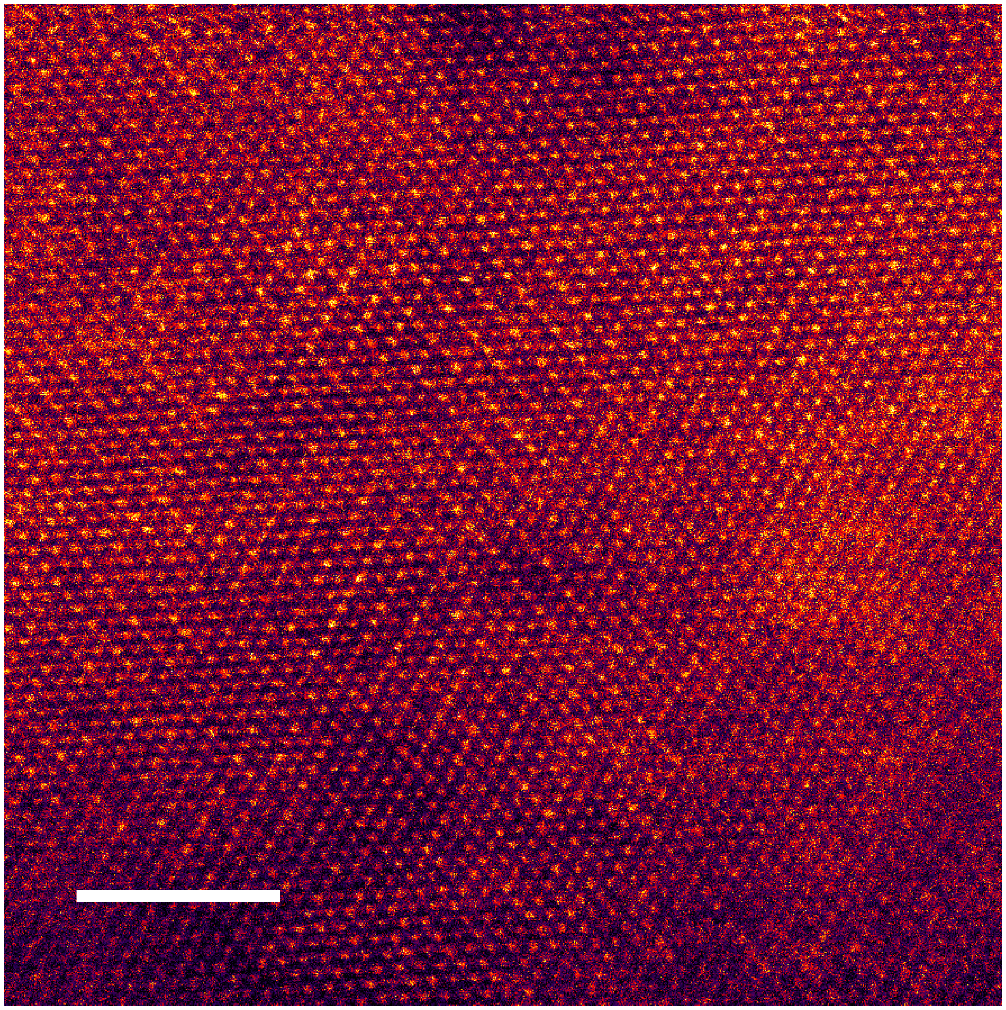

Figure S15: Raw atomic resolution image of the δ phase. Raw image from HAADF-STEM without filtering.

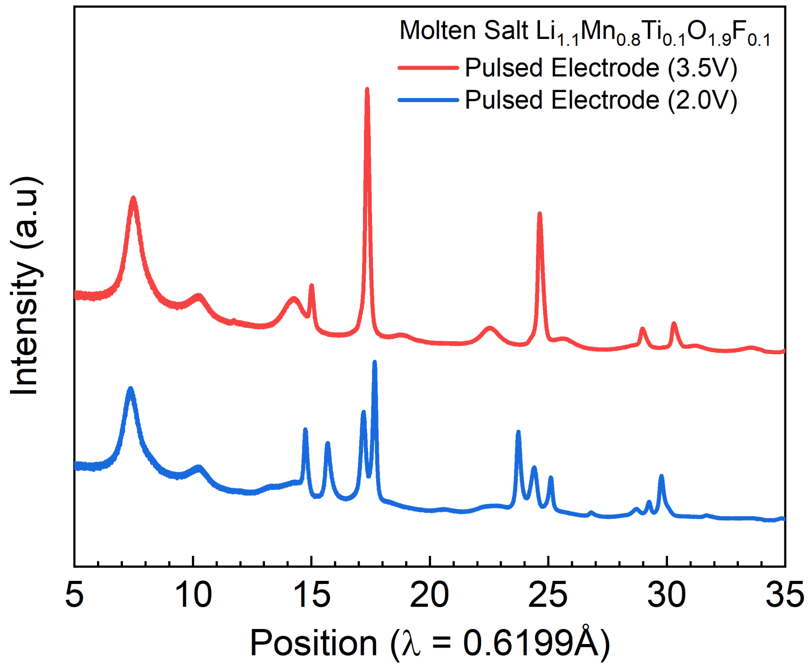

Figure S16: Observation of Jahn-teller distortion related peak splitting in synchrotron XRD. Observed peak splitting as a result of the symmetry reduction produced by the Jahn-Teller distortions in transformed molten salt synthesized material at the end of discharge to 2V.

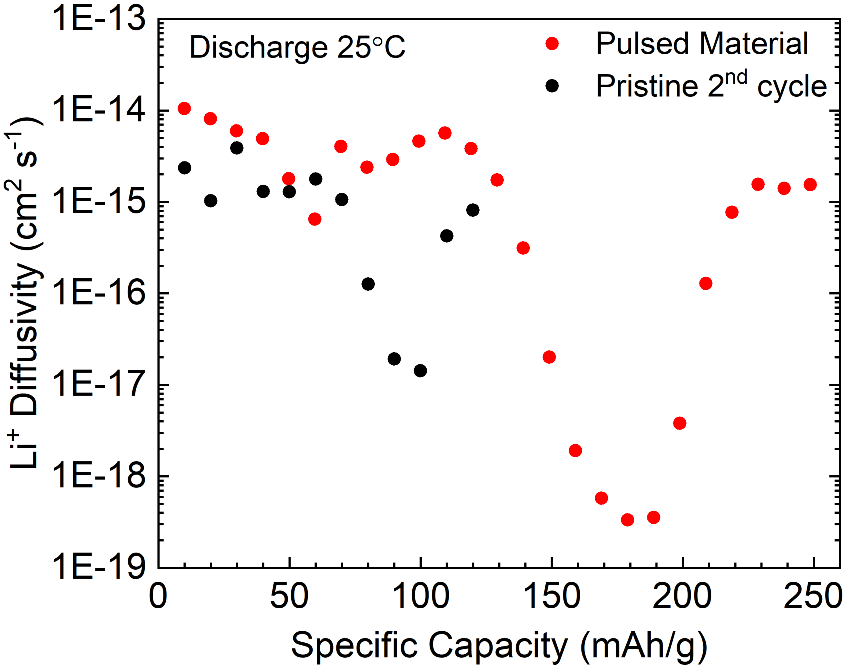

(a)

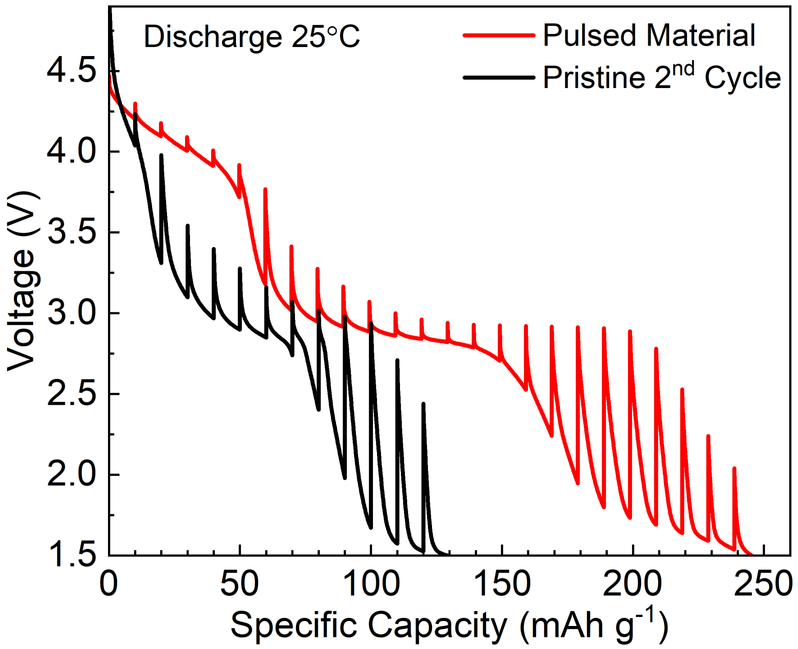

(b)

Figure S17: Galvanostatic intermittent titration technique (GITT) performed on molten salt synthesized material before and after pulsing. (a) Diffusivity estimates assuming a 2 micron particle diameter and (b) GITT curves during measurement.

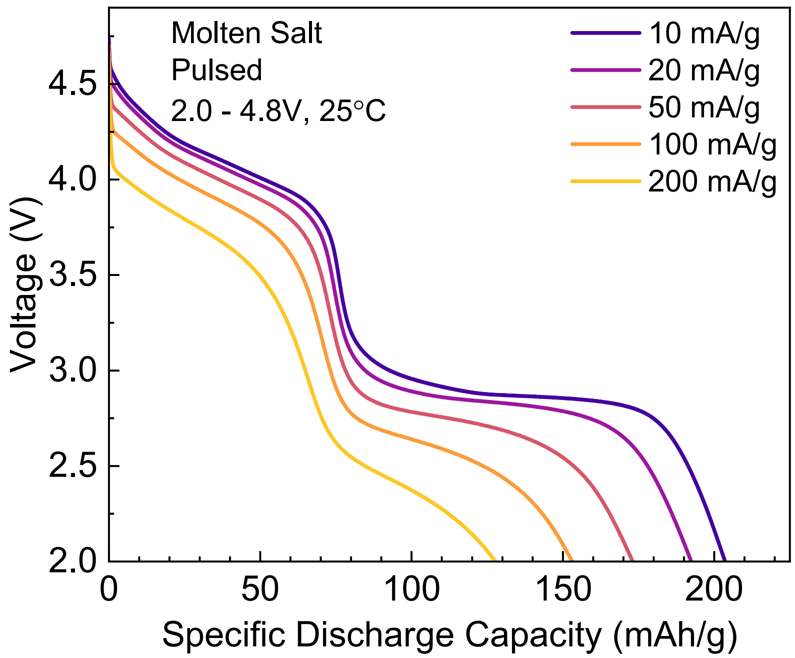

Figure S18: Rate capability of transformed molten salt material. Discharge voltage profiles for molten salt material after 80 pulses, charged at 10 mAh g^{-1}, and discharged at rates between 10 and 200 mAh g^{-1}.

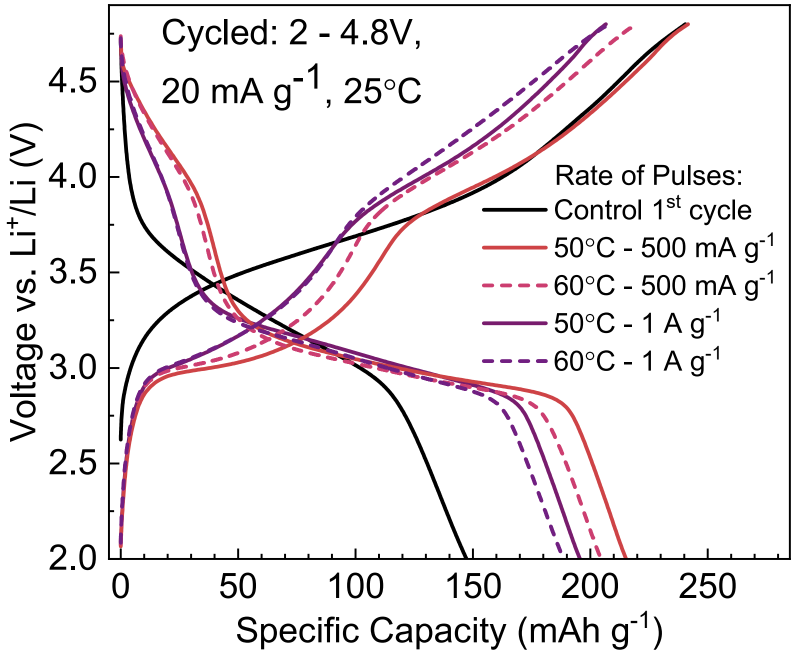

Figure S19: Effect of increased temperature on transformation. First cycle voltage curves for shaker-milled material pulsed at either 50°C or 60°C and 500 mA g^{-1} or 1 A g^{-1}.

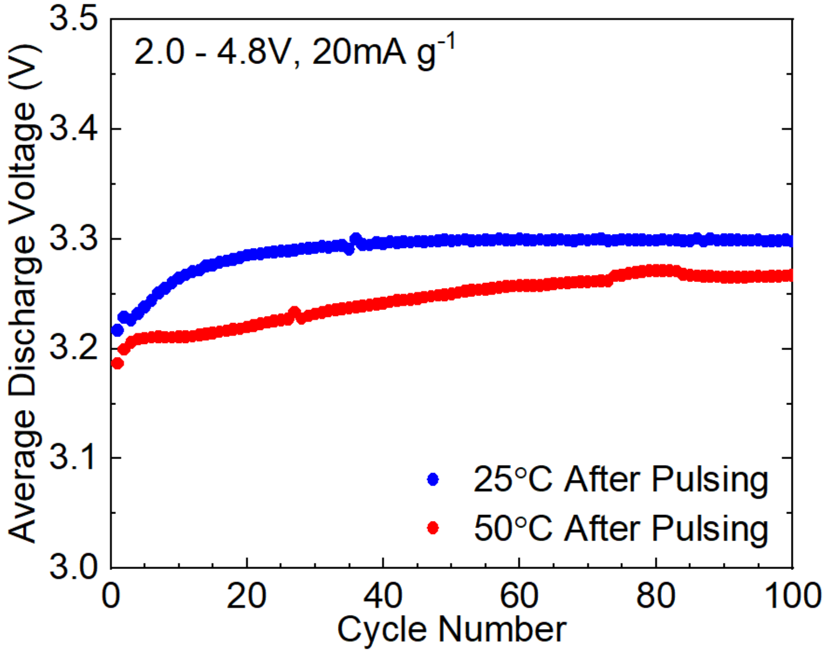

Figure S20: Voltage retention of pulsed MS sample in cycling. Average discharge voltage during the first 100 cycles for pulsed molten salt synthesized material at 25°C and 50°C.
